# Interplay Between Plasma Membrane Lipid Alteration, Oxidative Stress and Calcium-Based Mechanism for Extracellular Vesicle Biogenesis From Erythrocytes During Blood Storage

**DOI:** 10.3389/fphys.2020.00712

**Published:** 2020-07-03

**Authors:** Anne-Sophie Cloos, Marine Ghodsi, Amaury Stommen, Juliette Vanderroost, Nicolas Dauguet, Hélène Pollet, Ludovic D’Auria, Eric Mignolet, Yvan Larondelle, Romano Terrasi, Giulio G. Muccioli, Patrick Van Der Smissen, Donatienne Tyteca

**Affiliations:** ^1^CELL Unit and PICT Platform, de Duve Institute, Université catholique de Louvain, Brussels, Belgium; ^2^GECE Unit and CYTF Platform, de Duve Institute, Université catholique de Louvain, Brussels, Belgium; ^3^NCHM Unit, Institute of Neuroscience, Université catholique de Louvain, Brussels, Belgium; ^4^Louvain Institute of Biomolecular Science and Technology, Université catholique de Louvain, Louvain-la-Neuve, Belgium; ^5^Bioanalysis and Pharmacology of Bioactive Lipids Research Group, Louvain Drug Research Institute, Université catholique de Louvain, Brussels, Belgium

**Keywords:** lipid domains, membrane transversal asymmetry, reactive oxygen species, lipidomics, cell vesiculation, cholesterol, plasmatic acid sphingomyelinase, ceramide

## Abstract

The shedding of extracellular vesicles (EVs) from the red blood cell (RBC) surface is observed during senescence *in vivo* and RBC storage *in vitro*. Two main models for EV shedding, respectively based on calcium rise and oxidative stress, have been proposed in the literature but the role of the plasma membrane lipid composition and properties is not understood. Using blood in K^+^/EDTA tubes stored for up to 4 weeks at 4°C as a relevant RBC vesiculation model, we showed here that the RBC plasma membrane lipid composition, organization in domains and biophysical properties were progressively modified during storage and contributed to the RBC vesiculation. First, the membrane content in cholesterol and linoleic acid decreased whereas lipid peroxidation and spectrin:membrane occupancy increased, all compatible with higher membrane rigidity. Second, phosphatidylserine surface exposure showed a first rapid rise due to membrane cholesterol decrease, followed by a second calcium-dependent increase. Third, lipid domains mainly enriched in GM1 or sphingomyelin strongly increased from the 1st week while those mainly enriched in cholesterol or ceramide decreased during the 1st and 4th week, respectively. Fourth, the plasmatic acid sphingomyelinase activity considerably increased upon storage following the sphingomyelin-enriched domain rise and potentially inducing the loss of ceramide-enriched domains. Fifth, in support of the shedding of cholesterol- and ceramide-enriched domains from the RBC surface, the number of cholesterol-enriched domains lost and the abundance of EVs released during the 1st week perfectly matched. Moreover, RBC-derived EVs were enriched in ceramide at the 4th week but depleted in sphingomyelin. Then, using K^+^/EDTA tubes supplemented with glucose to longer preserve the ATP content, we better defined the sequence of events. Altogether, we showed that EV shedding from lipid domains only represents part of the global vesiculation mechanistics, for which we propose four successive events (cholesterol domain decrease, oxidative stress, sphingomyelin/sphingomyelinase/ceramide/calcium alteration and phosphatidylserine exposure).

## Introduction

Continuous change in membrane and membrane skeleton organization takes place during development from proerythroblasts to senescent red blood cells (RBCs). For instance, R1 reticulocytes undergo significant rearrangements in their membrane and intracellular components via several mechanisms including exosome release. Circulating R2 reticulocytes complete this maturational process, which involves additional loss of significant amounts of membrane and selected membrane proteins (Minetti et al., 2018, 2020). Membrane remodelling and vesicle formation also occur during RBC ageing (Ciana et al., 2017b). Bosman and coll. have proposed that generation of extracellular vesicles (EVs) constitutes a mechanism for the elimination of RBC membrane patches containing removal molecules, thereby postponing the untimely elimination of otherwise healthy RBCs (Willekens et al., 2008). Mechanistically, current knowledge indicates that part of the membrane skeleton is probably lost together with part of the lipid bilayer in a balanced way (Ciana et al., 2017a).

Those EVs are also released from RBCs *in vitro* upon storage partially expose phosphatidylserine (PS) at their outer membrane leaflet (Almizraq et al., 2018) leading to blood coagulation through thrombin generation (Rubin et al., 2013). RBC-derived EVs also promote excessive production of reactive oxygen species (ROS) and thereby exhortation of the “respiratory burst” in neutrophils (Jank and Salzer, 2011), which could be part of post-transfusional acute lung inflammation. Due to higher accessibility as compared to hemoglobin (Hb) entrapped in RBCs, Hb enclosed in RBC-derived EVs acts as a efficient nitric oxide scavenger, thereby reducing bioavailability of the latter as vasoregulator (Donadee et al., 2011; Said and Doctor, 2017).

Two main models for EV shedding from RBCs upon storage have been proposed. They are based on calcium rise and oxidative stress and lead to the destabilization of membrane:cytoskeleton anchorage followed by membrane loss through vesiculation (Alaarg et al., 2013; Tissot, 2013; Pollet et al., 2018). While the oxidative stress-based model is widely documented in the literature, the calcium-based model is still highly controversed for three main reasons (i) the origin of calcium accumulation is not elucidated; (ii) the contribution of this model to the EV release has been shown mainly through the use of calcium ionophores (Allan et al., 1980; Salzer et al., 2002; Nguyen et al., 2016); and (iii) the accumulation of calcium and the PS externalization, which were for a long time directly associated, are nowadays rather viewed as independent processes (Arashiki and Takakuwa, 2017). Discrepancies between studies could be reconciled if there are different types of EVs that are simultaneously or sequentially released by RBCs.

Surprisingly, the contribution of plasma membrane lipids in the vesiculation process is not understood. For instance, it is not known whether the budding of EVs could occur from specific regions of the plasma membrane and if some specific lipid domains could represent the starting point of the vesiculation process. Three types of submicrometric lipid domains have been identified at the outer plasma membrane leaflet of resting RBCs (i.e., RBCs that are not in the deformation process): (i) those mainly enriched in cholesterol (hereafter referred as cholesterol-enriched domains), (ii) those co-enriched in the ganglioside GM1, phosphatidylcholine and cholesterol (hereafter referred as GM1-enriched domains), and (iii) those co-enriched in sphingomyelin, phosphatidylcholine and cholesterol (hereafter referred as sphingomyelin-enriched domains) (Carquin et al., 2014, 2015, 2016; Conrard et al., 2018). During RBC deformation, cholesterol-enriched domains gather in high curvature areas, forming or stabilizing highly curved membrane regions. GM1- and sphingomyelin-enriched domains increase in abundance upon calcium influx and efflux respectively, thereby potentially participating to calcium exchanges, also essential for RBC deformation (Leonard et al., 2017, 2018; Conrard et al., 2018). Although those domains appeared to contribute to the RBC deformation process, their contribution to RBC vesiculation is not known. In support of the potential EV budding from specific regions of the RBC plasma membrane, the activity of the plasmatic acid sphingomyelinase (aSMase) has been proposed to be implicated in the biogenesis of EVs from RBCs during storage (Hoehn et al., 2017) but this is not proved and the link between ceramide production and membrane blebbing is still unclear.

The main goal of the present study was to evaluate whether the RBC plasma membrane lipid composition and organization could contribute to the RBC vesiculation process. To this aim, we used as main model RBCs stored in K^+^/EDTA tubes for two reasons. First, tubes are readily available by simple venipuncture and are less precious than RBC concentrates used for medical applications. Second, they allow to explore a significant amount of parameters upon a limited storage period of only 4 weeks, which is not possible on RBC concentrates that can be stored for up to 42 days before transfusion with limited storage lesions and vesiculation. We found that RBC vesiculation in K^+^/EDTA tubes was clearly accelerated as compared to RBC concentrates, mainly due to the rapid intracellular ATP drop. Nevertheless, the progressive rise of calcium and oxidative stress, as in RBCs upon senescence *in vivo* or upon storage in RBC concentrates (Pollet et al., 2018; Yoshida et al., 2019), supported the relevance of K^+^/EDTA tubes to explore the RBC vesiculation mechanisms. Thus, using this model, we determined the RBC membrane lipid composition, organization in lipid domains and biophysical properties during storage. To evaluate if lipid domains were lost by vesiculation, EVs isolated from RBCs were measured for their lipid content. Then, using K^+^/EDTA tubes supplemented with glucose, we could better define the sequence of events.

## Materials and Methods

### Blood Collection and Preparation

The study was approved by the Medical Ethics Committee of the University of Louvain, Brussels, Belgium. Blood from 13 adult healthy volunteers (11 women and 2 men), who gave written informed consent, was collected by venipuncture into K^+^/EDTA-coated tubes (except otherwise stated). Blood tubes were stored at 4°C for 0–30 days. From those storage days, storage time intervals covering a period of 4 days (except the first one which covered 3 days) were defined and were referenced in the whole manuscript as 0, 0.6, 1.2, 1.8, 2.4, 3.0, 3.6, and 4.2 storage weeks. To supplement blood with an additional energy source, 0.5 ml of Dulbecco’s Modified Eagle Medium (DMEM; LifeTechnologies) containing 4.5 g/l of glucose was added per 1 ml of blood in K^+^/EDTA-coated tubes directly after collection. Before experiments (except for EV isolation and measurement of aSMase activity), RBCs were isolated from other blood components by a 10-fold dilution in the adapted experimental medium (i.e., DMEM with or without glucose for RBC hemoglobin release, calcium, ATP and PS exposure measurements or Krebs-Ringer-Hepes (KRH) solution for ROS content determination; see below), washed twice by centrifugation at 200 *g* for 2 min and resuspended. This procedure allowed us to efficiently separate RBCs, as revealed by the absence of contamination of RBC preparations by platelets and white blood cells in our routine fluorescence imaging experiments.

### Chemical Treatments

All treatments were performed on washed isolated RBCs, except amitriptyline (AMI) which was applied on whole blood at 5 μM for 60 min at 37°C to inhibit the aSMase. To induce acute ATP depletion, RBCs were preincubated for 2 h in glucose-free DMEM (Life Technologies). ATP repletion was performed after RBC washing and resuspension in 4.5 g/l glucose-containing DMEM. To modulate the intracellular calcium content, RBCs were preincubated with 20 μM BAPTA-AM (Abcam): (i) for 15 min at 37°C, followed by a 60 min-reincubation at 37°C in the presence of Fluo-4 AM (see below), to measure its effect on the intracellular calcium content; or (ii) for 60 min at 37°C to determine EV abundance and PS exposure. To deplete membrane cholesterol, RBCs were preincubated with 0.9 mM methyl-β-cyclodextrin (mβCD; Sigma-Aldrich) for 30 min at 37°C. Cholesterol repletion was achieved with 7.5 and 15 μg/ml water-soluble cholesterol (Sigma-Aldrich) for 60 min at 37°C.

### RBC Scanning Electron Microscopy on Filters

Washed RBCs were pelleted, resupended and fixed in 0.1M cacodylate buffer containing graded concentrations (0.1, 0.5, and 1.5%) of glutaraldehyde for 5 min each. Fixed RBCs were then filtered on 0.4 μm polycarbonate (it4ip) filters using a syringe and washed by cacodylate buffer pushed gently through the syringe. Post-fixation was performed directly on the filters in the syringe in 1% OsO_4_ in 0.1M cacodylate for 2 h followed by extensive washing in 0.1M cacodylate and six times for 10 min in water. Samples were dissociated from the filter capsule and covered by a second filter in order to protect the sample during further processing, i.e., the dehydration and the critical point drying. Dehydration was performed in graded baths of ethanol (50, 60, 70, 80, 90, 95% for 10 min each, followed by 100% three times for 10 min) and critical point dried. Finally, samples were mounted on scanning electron microscopy stubs and sputtered with 10 nm gold. All samples were observed in the CM12 electron microscope with the SED detector at 80 kV.

### RBC Hemoglobin Release Measurement

Isolated RBCs were incubated at RT for 10 min into gradually hypotonic media prepared by dilution of DMEM with water. RBCs were then pelleted by centrifugation at 200 g for 2 min. RBC pellets were lysed in 0.2% Triton-X-100 and both supernatants and pellet lysates were assessed for Hb content by spectrophotometry (Packard SpectraCount Absorbance Microplate Reader) at 450 nm as oxy and deoxy forms of hemoglobin show main peaks of light absorption around 450 nm (Ratanasopa et al., 2015; Chung et al., 2020). For each medium, Hb in the supernatant was then expressed as ratio of the sum of Hb in the supernatant and the pellet. The osmolarity leading to 50% hemolysis (half-maximal effective hemolysis, EC50) was finally extrapolated using GraphPad Prism.

### RBC Calcium, ATP, ROS and Outer Plasma Membrane PS Measurements

All determinations were performed on isolated washed RBCs. Intracellular calcium was measured by fluorimetry using the Fluo-4 AM probe and normalized to the corresponding intracellular Hb content (detected by spectrophotometry as described above) as previously (Conrard et al., 2018). Such method generated only qualitative measurements as the Fluo-4 fluorescence signal is not a linear function of the calcium concentration (Kaestner et al., 2006). Alternatively, RBCs were labeled for 30 min at 37°C with Fluo-4 AM, washed and analyzed by flow cytometry (FACSVerse, BD Biosciences). Calcium exchanges upon RBC deformation were evaluated on RBCs spread onto a poly-L-Lysine (PLL)-precoated polydimethylsiloxane stretchable chamber (PDMS; Strex Inc) as in Conrard et al. (2018). Intracellular ATP content was measured using a chemiluminescence assay kit (Abcam). Luminescence produced during the reaction of the luciferase with luciferine in the presence of ATP was detected with a luminometer (GloMax Explorer Multimode Microplate Reader, Promega) and normalized to the Hb content. Intracellular ROS content and PS externalization were determined by flow cytometry. For ROS measurement, RBCs were labeled with 7.5 μM 2′,7′-dichlorodihydrofluorescein diacetate (H_2_DCFDA; Invitrogen) at 37°C for 20 min in KRH, washed and resuspended. For PS externalization, RBCs were incubated with Annexin-V FITC (Invitrogen; 25 μl for 5^∗^10^5^ RBCs) in DMEM at RT for 20 min. For all flow cytometry experiments, acquisition was performed at the FacsVerse with a medium flow rate and a total analysis of 10.000 events. The software FlowJo was used to determine upon storage (i) the Median Fluorescence Intensity (MFI) of whole RBC populations for ROS and calcium contents and PS exposure; and (ii) the percentage of PS-exposing RBCs by positioning the cursor at the edge of the labeled cell population at 0 week of storage.

### RBC Calpain Activity Measurement

RBCs were lysed in the buffer provided in the Calpain activity assay kit (Abcam). Twenty five μg proteins of the RBC lysates were then mixed with the “reaction buffer” and the calpain substrate, incubated for 1 h at 37°C and measured by fluorimetry (GloMax, Promega) at λ_exc_/λ_em_ of 400/505 nm.

### RBC Methemoglobin Determination

RBC lysates obtained through repeated freeze-thaw cycles were analyzed for methemoglobin (metHb) content following indications of a sandwich Elisa assay kit (LifeSpan Biosciences). Briefly, samples were added to the plate, incubated with a biotinylated detection antibody, washed and incubated with an avidin-conjugated Horseradish peroxidase, which in presence of a tetramethylbenzidine substrate induces blue color development. Addition of the stop solution induced color change into yellow and allowed metHb detection by spectrophotometry (SpectraCount, Packard) at 450 nm.

### RBC Spectrin Immunofluorescence

Isolated RBCs were immobilized onto PLL-precoated coverslips, permeabilized with 0.5% Triton X-100 for 3 min (to open the RBC and have access to the cytoskeleton overhanging the PLL-coated RBC membrane), fixed with 4% paraformaldehyde for 10 min and blocked with 1% bovine serum albumin (BSA) in phosphate buffer saline (PBS) for 30 min. RBCs were then labeled for 60 min at RT with an antibody against α/β-spectrin (Sigma; 1:100), washed and incubated for 2 h with the secondary antibody coupled to AlexaFluor488. All coverslips were finally mounted in Mowiol in the dark for 24 h and examined with a Zeiss LSM510 confocal microscope using a plan-Apochromat 63x NA 1.4 oil immersion objective. Spectrin membrane occupancy was quantified on confocal images using the Fiji software.

### RBC Membrane Cholesterol Content and Lipid Peroxidation Measurements

Cholesterol content was assessed using the Amplex Red cholesterol assay kit (Invitrogen) in the absence of cholesterol esterase (Grimm et al., 2005; Tyteca et al., 2010). Lipid peroxidation was determined with the Lipid Peroxidation (malondialdehyde, MDA) Assay kit (Abcam) applying the high sensitivity protocol (Tsikas, 2017). Both the cholesterol and the MDA levels were reported to the corresponding Hb content.

### RBC Membrane Fatty Acid Determination

RBC total lipids were extracted with chloroform/methanol/water (2:2:1.8; v:v:v) according to the Bligh and Dyer method. Nonadecanoic acid (C19:0; Sigma-Aldrich) was used as internal standard. Each sample was then dried under nitrogen and methylated at 70°C through a 1 h-incubation with 0.5 ml of 0.1 mol/l KOH in methanol, followed by a 15 min-incubation in 0.2 ml of 1.2 mol/l HCl in methanol. The fatty acid methyl esters (FAME) were then extracted by 1 ml hexane and separated by gaz chromatography (Schneider et al., 2012). The chromatograph (GC Trace-1310, Thermo Quest, Italy) was equipped with a RT2560 capillary column (100 m × 0.25 mm internal diameter, 0.2 μm film thickness; Restek) and a flame ionization detector (FID, Thermo Quest). The carrier gas used was H_2_ at constant pressure (200 kPa). The FID was continuously flowed by H_2_ (35 ml/min) and air (350 ml/min) and kept at a constant temperature of 255°C. The temperature program was as follows: an initial temperature of 80°C, which increased at 25°C/min up to 175°C, a holding temperature of 175°C during 25 min, a new increase at 10°C/min up to 205°C, a holding temperature of 205°C during 4 min, a new increase at 10°C/min up to 215°C, a holding temperature of 215°C during 25 min, a last increase at 10°C/min up to 235°C and a final holding temperature of 235°C during 10 min (Ferain et al., 2016).

### RBC Membrane Lipid Vital Imaging

To decorate endogenous membrane cholesterol, RBCs were labeled at RT with the mCherry-Theta toxin fragment at 0.6–0.7 μM and then spread onto PLL-coated coverslips, as in Conrard et al. (2018). Sphingomyelin, ceramide and GM1 ganglioside were analyzed through fluorescent BODIPY-sphingomyelin, -ceramide or -GM1 analogs inserted at the plasma membrane of RBCs spread onto PLL-coated coverslips, also as in Conrard et al. (2018). All samples were then placed upside down in Lab-Tek chambers (Fisher Scientific) filled with medium and observed with a Zeiss wide-field fluorescence microscope (Observer.Z1; plan-Apochromat 100X 1.4 oil Ph3 objective). For quantification, the number of lipid domains was assessed by manual counting on fluorescence images and reported to the hemi-RBC projected area determined with the Fiji software.

### Plasmatic aSMase Activity and pH Measurements

Whole blood diluted with PBS was laid down on Ficoll Paque Plus (GE Healthcare; 4:3 v/v) and centrifuged for 30 min at 400 *g* at RT to collect cell-free plasma. Plasma samples were increasingly diluted according to storage time to prevent interference with Hb released into plasma during hemolysis. Thereafter samples were processed according to indications of the Amplex Red Sphingomyelinase Assay Kit (Invitrogen). The plasmatic pH was determined using the GEM PREMIER 3500 (Instrumentation Laboratory; Croix-Rouge de Belgique).

### Blood EV Isolation and Analysis

Whole blood maintained at 4°C was centrifuged at 2000 *g* for 15 min. The plasma was recovered and centrifuged again at 2000 *g* for 15 min to pellet eventually remaining blood cells. The obtained plasma was diluted in sterile filtered PBS and centrifugated one additional time at 2000 *g* before ultracentrifugation at 20.000 *g* for 20 min at 4°C. The resulting EV pellet was resuspended in sterile PBS and the centrifugation step at 20.000 g was repeated. The final pellet was resuspended in 1 ml sterile PBS and used for electron microscopy, Nanoparticle tracking and/or flow cytometry analyses. For electron microscopy, isolated EVs were allowed to attach for 8 min onto PLL-coated coverslips. Coverslips were then washed with 0.1M cacodylate, fixed with 1% glutaraldehyde in 0.1M cacodylate and processed by scanning electron microscopy as for RBCs on filters (see above). Nanoparticle tracking analysis was realized with the Zetaview (Particle Metrix) by diluting the samples 5–5000-fold in sterile filtered PBS. For flow cytometry analysis, isolated EVs were successively labeled with 4 μM Carboxyfluorescein succinimidyl ester (CFSE, Invitrogen), 1 μg/ml anti-glycophorin A (GPA)-AlexaFluor647 antibodies (Bio-Rad) and 1 μg/ml anti-CD41-Phycoerythrin (PE) antibodies (BioLegend), each for 20 min at RT. Labeled EVs were analyzed with a FACSVerse (BD Biosciences) with a threshold set on CFSE fluorescence. If needed, samples were diluted for appropriate data acquirement.

### RBC-Released EV Purification and Analysis

EVs specifically released by RBCs were purified from other blood EVs by addition to 10^11^ EVs of 100 μl EasySep RBC Depletion Reagent magnetic beads coupled to anti-GPA antibodies (EasyPlate EasySep Magnet, StemCell). After 3 min, samples were split and charged on a 96-well plate which was then laid down on a magnetic plate (StemCell) for 3 min. Supernatants were discarded, the 96-well plate was separated from the magnet and the attached material was resuspended in sterile PBS. The deposit of the plate on the magnet and the resuspension of attached material were repeted and the purified material was finally collected into Eppendorfs. Purity of preparations was then analyzed by western blotting using 4–15% sodium dodecylsufalte-polyacrylamide gel electrophoresis (SDS-PAGE; Bio-Rad) and antibodies against CD41 (Abcam, 1:2000) and GPA (Abcam, 1:500), followed by incubation with HRP-conjugated secondary antibodies and revelation by chemiluminescence (SuperSignal west pico/femto chemiluminescent substrate, Thermo Fisher Scientific) with the Fusion Solo S from Vilber.

### RBC-Released EV Lipid Quantification

Lipid content of sorted EVs was characterized by liquid chromatography–mass spectrometry (LC-MS). Lipid species were analyzed after liquid/liquid extraction and solid phase extraction purification in the presence of internal standards. Lysophospholipids and phospholipids were analyzed on a LC-coupled tandem quadrupole (Xevo-TQS from Waters). A Kinetex LC-18 (150 × 4.6 mm, 5.0 μm) column (Phenomenex) and a gradient between phase A [MeOH-ACN (9:1, v/v) 75%, H_2_O 25% containing 5 mM ammonium acetate], phase B [MeOH-ACN (9:1, v/v) containing 5 mM ammonium acetate] and phase C (IpOH containing 5 mM ammonium acetate) were used. The gradient (400 μl/min) increased linearly from 100% A to 100% B in 15 min, and after 10 min from 100% B to 70% B – 30% C over 5 min. This was maintained over 30 min before requilibrating the system. The lipids were analyzed in negative mode using an ESI probe. Sphingomyelin species were analyzed using a LTQ-Orbitrap mass spectrometer coupled to an Accela LC system (from Thermo Fischer Scientific) using the same chromatographic system as described above. Those species were analyzed in negative mode using an ESI probe (Guillemot-Legris et al., 2016). Ceramides and dihydroceramides were analyzed using a LTQ-Orbitrap mass spectrometer coupled to an Accela LC system (from Thermo Fischer Scientific) as in Mutemberezi et al. (2016). A Poroshell 120 LC-18 (150 × 4.6 mm, 4.0 μm) column (Agilent) and a gradient between phase A (MeOH-H_2_O (75:25, v/v) containing 0.1% of acetic acid) and phase B (MeOH containing 0.1% acetic acid) were used. The gradient (400 μl/min) increased linearly from 100% A to 100% B over 15 min and was held at 100% for an additional 75 min before requilibrating the system. These lipids were analyzed in positive mode using an APCI probe. For all lipid species, the relative quantification was based on the ratio of area under the curve (AUC) of the lipid of interest and the AUC of the respective internal standard.

### Data Presentation and Statistical Analyses

Data are expressed as means ± SEM when the number of independent experiments was ≥3 or as means ± SD if *n* ≤ 2. For kinetics, the storage time needed to induce half-effect was determined with GraphPad Prism (EC50). Statistical analyses were applied only if three independent experiments were available. For kinetics, the statistical signification of the differences between data before and after the EC50 was determined with the Mann–Whitney *U* test (only when *n* ≥ 3). For the effect of pharmacological agents, the Wilcoxon signed rank test or Friedman test followed by Dunn’s multiple comparisons test were used for paired data whereas the Kruskal–Wallis test followed by Dunn’s multiple comparisons test was used for unpaired data. ns, not significant; ^∗^*p* < 0.05; ^∗∗^*p* < 0.01; ^∗∗∗^*p* < 0.001; ^****^p < 0.0001.

## Results

### Upon Storage in K^+^/EDTA Tubes for Up to 4 Weeks at 4°C, RBCs Rapidly Lose Biconcavity and Energy and Become More Fragile

**FIGURE 1:**
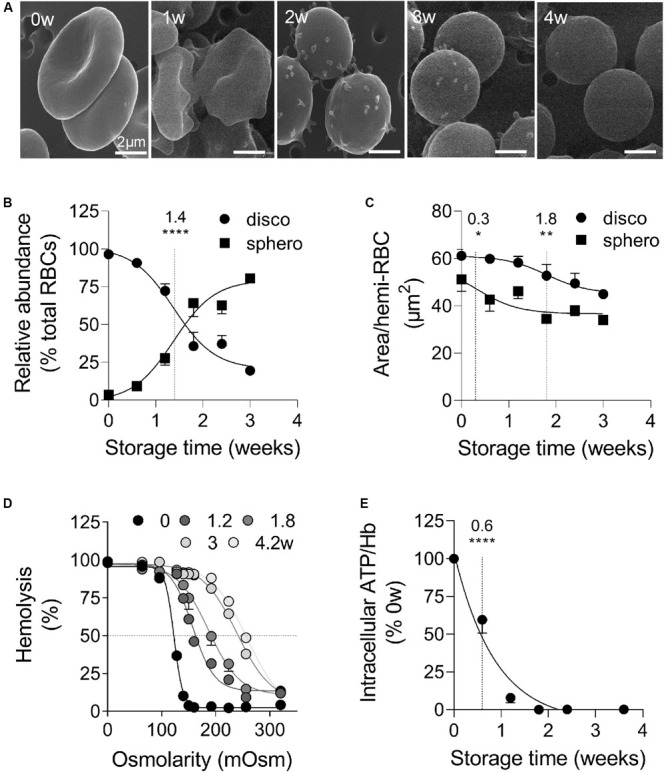
During storage in K^+^/EDTA tubes at 4°C, RBCs rapidly lose biconcavity and energy, become more fragile and form surface vesicles. RBCs stored for 0 to 4 weeks (w) in K^+^/EDTA tubes at 4°C were isolated and analyzed for morphology **(A,B)**, size **(C)**, fragility **(D)**, and intracellular ATP content **(E)**. The time needed to reach 50% effect and the corresponding statistical analysis (Mann–Whitney *U* test) are indicated above the vertical dotted line. **(A)** Scanning electron microscopy images of RBCs on filters. RBCs were fixed in increasingly concentrated glutaraldehyde solutions, deposited on filters, post-fixed with osmium tetroxide, covered with gold and observed by electron microscopy. Representative images of 1 experiment including 3 preparations per storage time. **(B,C)** Abundance and surface area of spherocytes *vs.* discocytes. RBCs were immobilized on poly-L-lysine (PLL)-coated coverslips, imaged and classified into two categories, the discocytes (disco; circles) and the spherocytes (sphero; squares), which are smaller and possess higher circularity. In **(B)**, data are means ± SEM of 7 independent experiments where at least 100 RBCs were analyzed. In **(C)**, data are means ± SEM of 4 independent experiments where at least 150 RBCs were analyzed. **(D)** RBC osmotic fragility. RBCs were incubated at RT in media of decreasing osmolarity. After centrifugation, the hemoglobin (Hb) content in the supernatant and in the pellet were measured by spectrophotometry at 450 nm. The horizontal dotted line indicates the medium osmolarity at which 50% of the RBCs were lysed. Data are means ± SD of 1 experiment representative of 2. **(E)** Intracellular ATP content. RBCs were lysed and incubated with firefly luciferase and luciferin. The intensity of the light emitted following the oxidation of luciferin to oxyluciferin by luciferase in the presence of ATP was then measured by luminescence. The intracellular ATP concentration was normalized on the Hb content and then expressed in % of fresh RBCs (i.e., 0 weeks of storage). Data are means ± SEM of 4 independent experiments. *p < 0.05; **p < 0.01; ****p < 0.0001.

RBCs stored for up to 4 weeks at 4°C sustained morphological transformation from biconcave discocytes to smaller and rounded spherocytes, as revealed by scanning electron microscopy (Figure 1A). The abundance of discocytes *vs.* spherocytes was in favor of the latter from 1.4 weeks of storage (Figure 1B) and was accompanied by membrane area loss (Figure 1C). The high proportion of spherocytes at the beginning of the storage period (Figure 1B) probably mainly resulted from RBC swelling. Moreover, transition to spherocytes was accompanied by the budding of EVs, mostly visible at 2–3 weeks of storage (Figure 1A), and by increased RBC fragility, fresh and 4.2 weeks-stored RBCs exhibiting 50% hemolysis in 120 and 260 mOsm media respectively (Figure 1D). The intracellular ATP decreased by ∼50% after 0.6 weeks and was totally depleted from 1.8 weeks (Figure 1E). Of note, ATP levels in fresh RBCs were similar whatever the anticoagulant used for the venipuncture (Supplementary Figure S1A) and the ATP drop was not biased by normalization with the global Hb content which was largely preserved during the storage period (Supplementary Figure S2A). Altogether those data indicated that RBC storage in K^+^/EDTA tubes induced morphological and biochemical storage lesions, including RBC vesiculation.

### Upon Storage in K^+^/EDTA Tubes for Up to 4 Weeks at 4°C, RBCs Release a High Amount of Extracellular Vesicles

**FIGURE 2:**
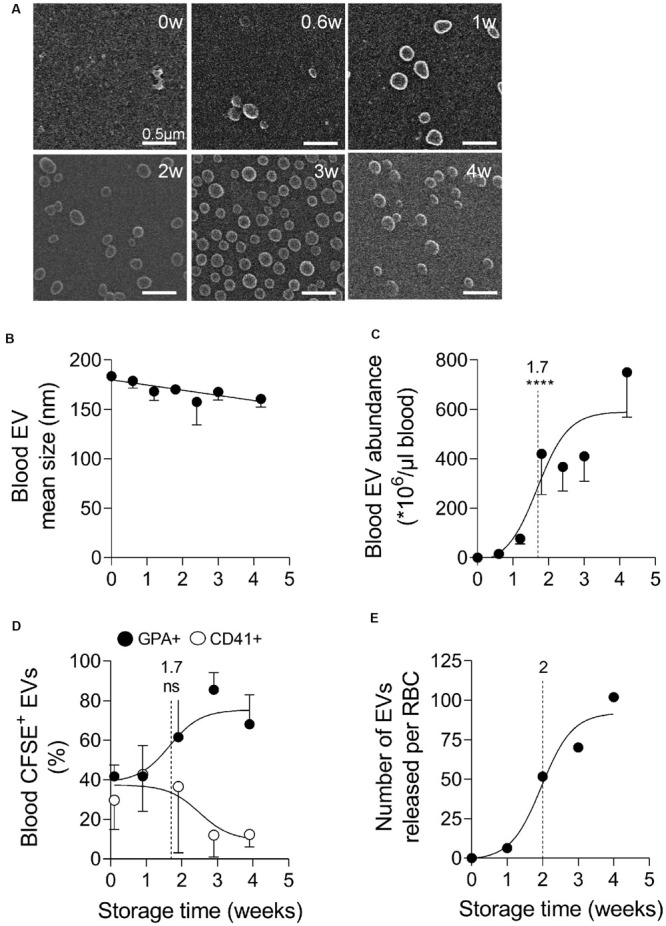
Blood extracellular vesicles increase in abundance upon storage and originate from both platelets and RBCs in the first 2 weeks of storage, then mainly from RBCs. Extracellular vesicles (EVs), isolated by ultracentrifugation from plasmas obtained from K^+^/EDTA tubes stored at 4°C for the indicated times, were analyzed for morphology **(A)**, size **(B)**, abundance **(C)**, and cellular origin **(D,E)**. The time needed to reach 50% effect and the corresponding statistical analysis (Mann–Whitney *U* test) are indicated above the vertical dotted line. **(A)** EV morphology evidenced by electron microscopy. Isolated EVs were immobilized on PLL-coated coverslips, fixed with glutaraldehyde, post-fixed with osmium tetroxide, gold-labeled and observed by electron microscopy. Representative images from 1 experiment. **(B,C)** EV size and concentration determined by nanoparticle tracking analysis using the ZetaView. In **(B)**, EV size expressed in nm. In **(C)**, number of EVs/μl of blood determined as follows. The number of EVs/μl of liquid in which EVs were suspended was determined by the Zetaview and then normalized by the factor between the volume of plasma used for EV isolation and the volume of plasma contained in 1 μl of blood (i.e., 0.6 μl). Data are means ± SEM of 5 independent experiments. **(D)** Proportion of blood EVs produced by RBCs and platelets determined by flow cytometry. Isolated EVs were labeled with fluorescent carboxyfluorescein succinimidyl ester (CFSE) and anti-glycophrorin A (anti-GPA-AlexaFluor647; black circles) and -CD41 (anti-CD41-PE; white circles) antibodies to respectively label EVs originating from RBCs and platelets and then analyzed by flow cytometry. Results are expressed as % of GPA (RBCs) or CD41 (platelets) positive CFSE-labeled EVs. Mean ± SEM of 4 independent experiments. **(E)** Estimation of the abundance of EVs released per RBC based on the number of EVs per μl of blood **(C)** and the proportion of EVs produced by RBCs **(D)**. ns, not significant; ****p < 0.0001.

As RBCs stored for up to 4 weeks exhibited vesicles at their surface (Figure 1A), we next isolated those vesicles from the plasma by ultracentrifugation and analyzed them for morphology, size, abundance and cell origin. Blood EVs were characterized by a round to slightly elongated morphology (Figure 2A) and a relatively homogenous size ranging from 160 to 180 nm (Figure 2B). Their abundance strongly rised during storage, showing a half-maximal increase at 1.7 weeks and a 600-fold increase after 4 weeks (Figure 2C). Since platelets and megakaryocytes are generally considered as main EV source (at least in fresh blood; Italiano et al., 2010) we next determined the relative proportion of EVs released by platelets and RBCs upon labeling with CFSE (a general EV marker) and fluorescent antibodies directed against GPA and CD41 (to decorate respectively RBC- and platelet-released EVs) and flow cytometry analysis. At time 0, RBC- and platelet-produced EVs were detected in equivalent proportions (Supplementary Figure S3 and Figure 2D). The half-maximal increase of EVs produced by RBCs was seen at 1.7 weeks, i.e., exactly at the same time than the half-maximal increase of blood EVs (Figure 2C). Based on the number of EVs per μl of blood and the proportion of EVs produced by RBCs *vs.* platelets, we next calculated the number of EVs produced per RBC during storage (Figure 2E). While only five EVs were released per RBC after 1 week of storage, this number rised up to 50, 70, and 100 at 2, 3, and 4 weeks.

### The RBC Methemoglobin Content and the Calpain Activity Increase Faster Than Calcium and ROS Intracellular Contents Upon Storage

**FIGURE 3:**
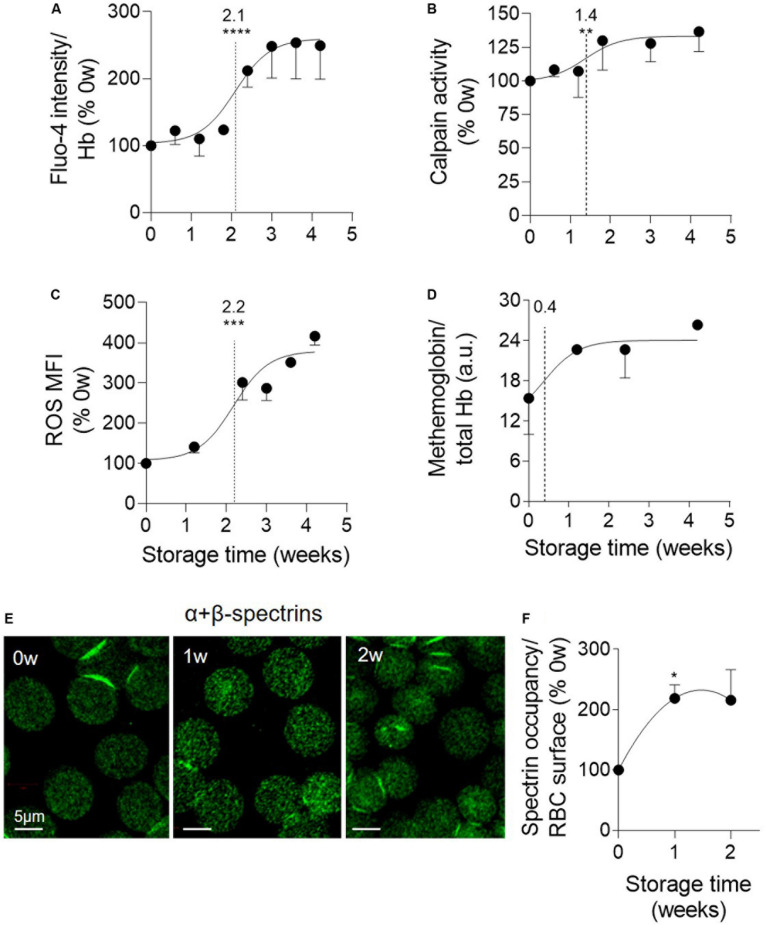
Oxidized hemoglobin and spectrin density rapidly increase upon storage while calpain activity increase and accumulation of calcium and reactive oxygen species take place later. RBCs stored for the indicated times at 4°C in K^+^/EDTA tubes were isolated and analyzed for intracellular calcium **(A)**, active calpain **(B)**, reactive oxygen species (ROS; **C**), methemoglobin (metHb) content **(D)** and spectrin occupancy at the RBC surface **(E,F)**. The time needed to reach 50% effect and the statistical analysis (Mann–Whitney *U* test) are indicated above the vertical dotted line [except in **(F)** in which the comparison was done *vs.* the 0 week]. **(A)** RBC calcium content. RBCs were incubated with the non-fluorescent Fluo-4 AM and then in Fluo-4 AM-free medium to allow for probe de-esterification and calcium binding, generating fluorescent Fluo-4 measured at 494 nm. Data were normalized on the Hb content and expressed in % of 0 weeks of storage. Means ± SEM of 7 independent experiments. **(B)** μ-calpain activity. Lysates of isolated RBCs were incubated with the substrate of calpain which emits fluorescence at 505 nm upon cleavage. Data are expressed as % of 0 weeks of storage and are means ± SEM of 6 independent experiments. **(C)** RBC ROS content. RBCs were labeled with H_2_DCFDA, which is transformed into fluorescent 2,7-dichlorofluorescein (DCF) after de-esterification and interaction with intracellular ROS, and then analyzed by flow cytometry. Median fluorescence intensity (MFI) as % of 0 weeks of storage. Means ± SEM of 3 independent experiments. **(D)** MetHb determination. RBCs were assessed for metHb concentration by a sandwich ELISA. Data were then normalized to the total Hb content. Means ± SD of 2 independent experiments. **(E,F)** Membrane:spectrin occupancy. RBCs were immunolabeled for pan-spectrin, analyzed by confocal microscopy **(E)** and quantified for spectrin occupancy per RBC surface **(F)**. Data were expressed as % of 0 weeks of storage and are means ± SEM of 3 independent experiments. *p < 0.05; **p < 0.01; ***p < 0.001; ****p < 0.0001.

The next step was to determine whether the calcium rise and the oxidative stress could contribute to the RBC vesiculation in K^+^/EDTA tubes as in RBCs upon senescence or upon storage in RBC concentrates. The RBC calcium content increased by half after 2.1 weeks and showed a ∼2.5-fold increase after 4 weeks of storage (Figure 3A). Of note, neither the EDTA present in the tubes nor data normalization by the global Hb content appeared to affect the intracellular calcium content (Supplementary Figures S1B,C, S2B). The activity of the calcium-dependent calpain was only slightly but significantly increased with a half-maximal effect at 1.4 weeks of storage (Figure 3B). In contrast, the RBC content in free ROS highly increased during the storage period, as reflected by the strong rise of the median fluorescence intensity of the RBC population labeled with H_2_DCFDA. The half-maximal effect was detected at 2.2 weeks (Figure 3C and Supplementary Figure S4A). Accordingly, metHb (the oxidized form of Hb) also increased, exhibiting a very rapid half-maximal effect at 0.4 weeks and a maximal increase from 1.2 weeks of storage (Figure 3D). Those data indicated that the calcium- and the oxidative-based models were both relevant to RBC storage in K^+^/EDTA tubes.

### Spectrin:Membrane Occupancy and Membrane Rigidity Increase Upon Storage

To then analyze the RBC cytoskeleton:membrane interaction, the α- and β-spectrin tetramers were immunolabeled and vizualized by confocal microscopy. A significant increase of the spectrin occupancy per RBC area was already visible after the first week of storage and maintained thereafter (Figures 3E,F). To decipher whether membrane:cytoskeleton alteration could in turn modify membrane rigidity, confocal imaging with Laurdan, a fluorescent tracer of membrane lipid order (Golfetto et al., 2013), was performed. This analysis revealed an increased membrane rigidity of discocytes, but not of spherocytes after 2 weeks of storage (Supplementary Figure S5), suggesting that the loss of EVs allowed to partially restore membrane lipid order. The increased membrane rigidity, which was confirmed by atomic force microscopy (data not shown), did not appear to result from changes of the plasmatic pH which only slightly decreased during the 4-week storage period (Supplementary Figure S6).

### Membrane Cholesterol and Linoleic Acid Decrease During Storage While Lipid Peroxidation Slightly Increases

**FIGURE 4:**
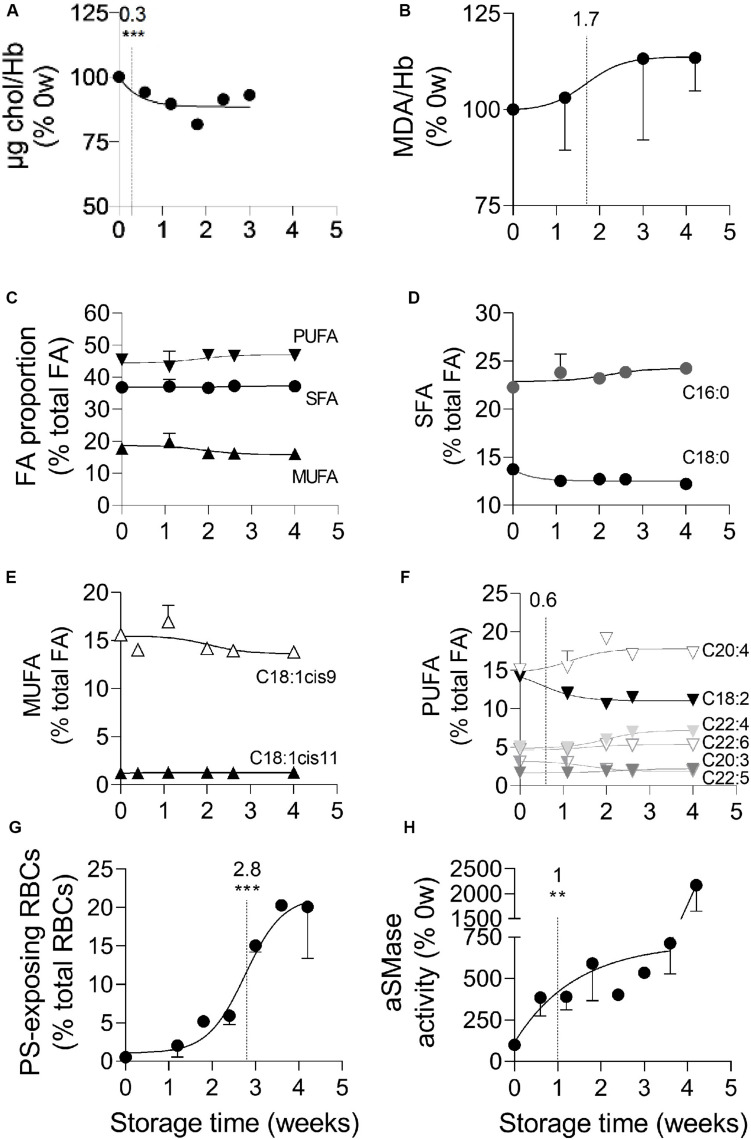
Cholesterol and linoleic acid (C18:2) decreases, plasmatic acid sphingomyelinase activation, lipid peroxidation and PS surface exposure successively take place at the RBC membrane during storage. RBCs isolated from K^+^/EDTA tubes stored at 4°C were analyzed for their membrane content in cholesterol (chol, **A**), peroxidized lipids **(B)**, fatty acids (FA; **C–F**) and external phosphatidylserine (PS; **G**) while isolated plasmas were assessed for the plasmatic acid sphingomyelinase (aSMase) activity **(H)**. The time needed to reach 50% effect and the corresponding statistical analysis (Mann–Whitney *U* test) are indicated above the vertical dotted line. **(A)** Chol content. Chol was quantified based on successive enzymatic reactions leading to the production of H_2_O_2_ which allows the transformation of non–fluorescent Amplex Red into fluorescent resofurin measured at 585 nm. Data were normalized to the Hb content and expressed in % of 0 week of storage. Means ± SEM of 4 independent experiments. Error bars were included in the symbols. **(B)** Malondialdehyde (MDA) content. Lysates of isolated RBCs were incubated with thiobarbituric acid (TBA) which reacts with free MDA, one of the end products of lipid peroxidation, to form a fluorescent adduct measured at 553 nm. Data were normalized to the Hb content and expressed in % of 0 weeks of storage. Means ± SD of 2 independent experiments. **(C–F)** FA composition. Total lipid extraction from isolated RBCs was followed by methylation and extraction of FA methyl esters and analysis by gas chromatography. All data are means ± SD of triplicates from 1 representative experiment out of 2 and are expressed as % of total FAs. **(C)** Proportion of saturated fatty acids (SFA), mono- (MUFA) and poly-unsaturated fatty acids (PUFA). **(D)** Relative proportion of the two major SFAs (C16:0 and C18:0). **(E)** Relative proportion of the two major MUFAs (i.e., C18 with one double bond either on position 9 or 11). **(F)** Relative proportion of the major PUFAs (chains of 18–22C and 2–6 double bonds). **(G)** Exposure of PS at the RBC surface. RBCs were labeled with Annexin-V coupled to FITC and then analyzed in flow cytometry. The % of Annexin V-FITC positive RBCs was determined by positioning the cursor at the edge of the labeled cell population at 0 weeks of storage. Data are means ± SEM of 3 independent experiments. **(H)** aSMase activity. The activity of aSMase in isolated plasmas was measured thanks to successive enzymatic reactions leading to the formation of a fluorescent product, the resorufin, measured at 585 nm. Means ± SEM of 5 independent experiments. **p < 0.01; ***p < 0.001.

We then determined whether the increased membrane rigidity resulted exclusively from the stronger membrane:spectrin occupancy or also from alterations of membrane lipid composition and organization. We started by analyzing the RBC membrane lipid composition, giving a particular attention to cholesterol and fatty acid (FA) unsaturation, both involved in the regulation of membrane fluidity (Arisawa et al., 2016; Subczynski et al., 2017), and to peroxidized lipids, since ROS were strongly increased upon storage. The membrane cholesterol content decreased slightly but very rapidly, with a half-maximal effect at 0.3 weeks of storage (Figure 4A). A slight rise of lipid peroxidation was also detected, with a half-maximal increase at 1.7 weeks (Figure 4B). None of those modifications was influenced by data normalization by global Hb content (Supplementary Figures S2C,D). To then evaluate the relative proportion of saturated *vs.* (poly)unsaturated FA, gas chromatography was used. A total of 42 FA belonging to phospholipids, sphingolipids and neutral lipids or consisting in free FA were detected and thereafter classified into three groups, saturated (SFA), monounsaturated (MUFA) and polyunsaturated FA (PUFA). The relative abundance of FA on fresh RBCs was in good agreement with (Rise et al., 2007). No major changes in the proportion of those three groups was observed during the storage (Figure 4C). The proportion of the two major SFA (C16:0 and C18:0) and MUFA (C18:1cis9 and C18:1cis11) was also largely maintained (Figures 4D,E). Among PUFA, the C18:2c9c12 (or linoleic acid) decreased at the benefit of long chain PUFA (such as C20:4 and C22:4), with a half-time effect at 0.6 weeks (Figure 4F). In conclusion, the RBC membrane lipid composition was altered upon blood storage, exhibiting decreased cholesterol and linoleic acid contents and increased lipid peroxidation.

### PS Surface Exposure Rapidly Increases Upon Storage

To next evaluate the membrane transversal asymmetry, PS surface exposure was determined by flow cytometry upon RBC labeling with fluorescent Annexin V. Whereas the mean fluorescence intensity did not increase upon storage (Supplementary Figure S4B), the proportion of PS-exposing RBCs already increased by ∼5-fold during the first 2 weeks of storage, showed a half-maximal significant effect at 2.8 weeks and then continued to increase throughout the entire storage period (Supplementary Figure S4C and Figure 4G). Thus, as compared to calcium rise (see Figure 3A), the increase of abundance of PS-exposing RBCs showed a delayed half-maximal effect but started more rapidly.

### The Plasmatic aSMase Activity Increases in Two Stages Upon Storage

To assess whether membrane lateral asymmetry could also be affected upon storage, we evaluated the activity of the aSMase, which is responsible for the transformation of sphingomyelin into ceramide and was proposed to induce RBC vesiculation (Dinkla et al., 2012; Pollet et al., 2018). The aSMase activity increased in a strong and fast manner, with a half-maximal effect at 1 week of storage, followed by a second increase after 4 weeks of storage (Figure 4H).

### Sphingomyelin- and GM1-Enriched Domains Increase in Abundance Upon Storage Whereas Cholesterol- and Ceramide-Enriched Domains Decline

**FIGURE 5:**
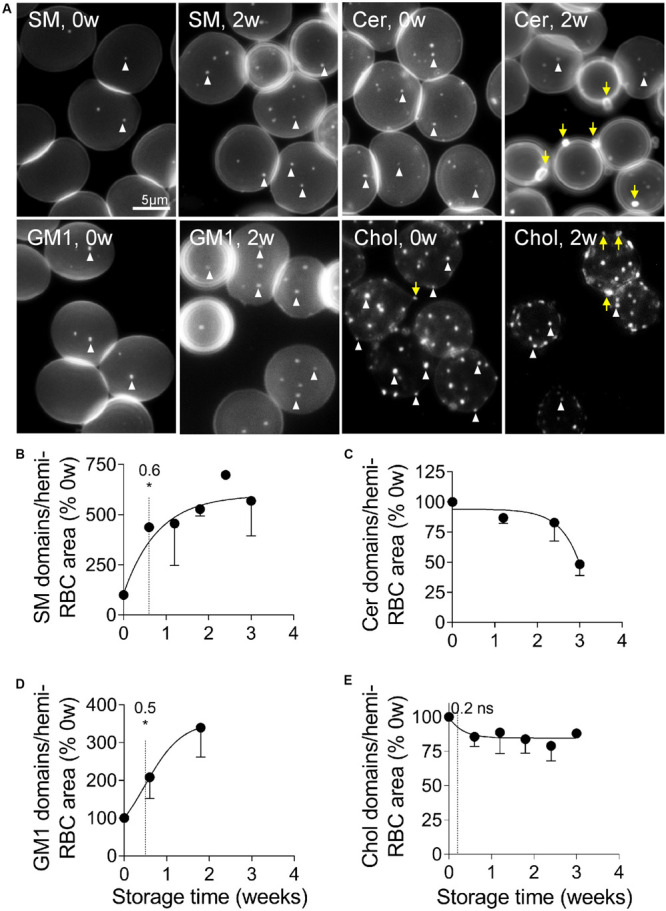
Sphingomyelin- and GM1-enriched domains increase in abundance upon storage whereas those enriched in ceramide and cholesterol decrease. RBCs isolated from K^+^/EDTA tubes stored at 4°C for the indicated times were either immobilized on PLL-coated coverslips and then labeled with fluorescent analogs of sphingomyelin (SM; **A,B**), ceramide (cer; **A,C**) or ganglioside GM1 (GM1; **A,D**); or first labeled with the fluorescent Theta toxin specific to endogenous cholesterol and then immobilized on PLL-coated coverslips (chol; **A,E**). All coverslips were then directly observed by fluorescence microscopy. **(A)** Representative images of lipid domains on RBCs stored for 0 and 2 weeks. White arrowheads, lipid domains; yellow arrows, lipid-enriched EVs. **(B–E)** Quantification. 150–300 RBCs per storage time and per lipid were counted for the number of domains. This number was then divided by the average RBC surface area and expressed as % of 0 weeks of storage. Data are means ± SD of 2 experiments in **(C)** and means ± SEM of at least 3 independent experiments in **(B,D,E)**. The time needed to reach 50% effect and the corresponding statistical analysis (Mann–Whitney *U* test) are indicated above the vertical dotted line. ns, not significant; *p < 0.05.

The above observation prompted us to evaluate by fluorescence microscopy the lateral distribution of sphingomyelin and ceramide at the RBC surface. This analysis was limited to the three first weeks of storage because the loss of membrane area per hemi-RBC above this period becomes significant (see Figure 1C) and would therefore have required careful adaptation of the labeling procedure to avoid toxicity. As expected from our previous data (Conrard et al., 2018), sphingomyelin clustered into well-defined submicrometric domains associated with the center of the fresh RBCs, which correspond to the RBC low curvature areas (Leonard et al., 2017, Figure 5A, 0w). Quite surprisingly, the abundance of those domains increased strongly (by six fold) and rapidly upon storage (Figure 5B, EC50, 0.6w; Figure 5A, SM, 2w), in a kinetics similar to that observed for the aSMase activity (see Figure 4H). Ceramide also clustered into domains well-visible on fresh RBCs, but in contrast to the sphingomyelin-enriched domains, they decreased upon storage (Figures 5A,C). We then analyzed the two other submicrometric domains present at the RBC surface, i.e., those mainly enriched in GM1 and those mainly enriched in cholesterol (Conrard et al., 2018). Like sphingomyelin-enriched domains, GM1-enriched domains increased in abundance upon storage, with a similar half-time effect (Figures 5A,D). In contrast, cholesterol-enriched domains very rapidly (EC50, 0.2) but slightly decreased (Figures 5A,E). In conclusion, whereas sphingomyelin- and GM1-enriched domains increased in abundance upon storage, those enriched in ceramide and cholesterol instead decreased. The next question was whether those modifications resulted from domain loss by vesiculation, which was investigated through the determination of the lipid content of RBC-derived EVs.

### RBC-Derived EVs Are Enriched in Ceramide, Lysophosphatidylinositol and Lysophosphatidylglycerol Species at the End of the Storage but Are Nearly Depleted in Their Precursors

**FIGURE 6:**
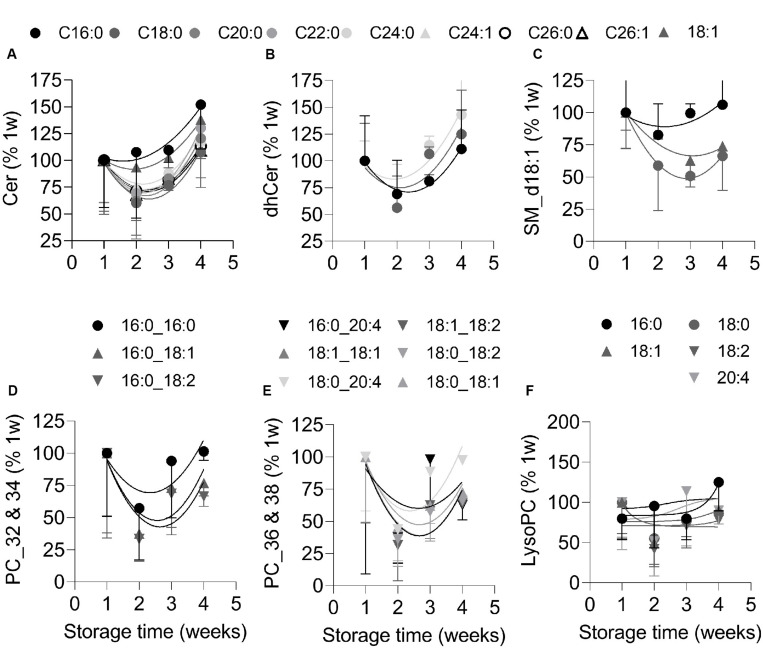
RBC-derived EVs are enriched in ceramide and dihydroceramide species at the end of storage but nearly depleted in sphingomyelin and phosphatidylcholine species. RBC-released EVs were isolated from total blood EVs by immunopurification and analyzed by LC-MS for their content in: **(A,B)** ceramide (cer) and dihydroceramide (dhcer) species; **(C)** SM d18:1 species; and **(D–F)** phosphatidylcholine (PC; presented in two graphs according to the length of their carbon chains) and lysoPC species. Each curve depicts one lipid species; circle, saturated lipid; triangle, lipid containing at least one MUFA; inverted triangle, lipid containing at least one PUFA; black to light gray and white colors, short to long carbon chains. Notice that most of the SM species (e.g., 16:1) and some PC species (e.g., 16:0_18:0) were at the limit of detection. Representative experiment out of 2. All data were expressed as % of the EVs stored for 1 week and are means ± SD of 4 replicates.

To analyze the lipid content of EVs originating from RBCs without contamination by those produced by platelets (see Figure 2D), the former were isolated by immunopurification. Purity of preparations was confirmed by Western Blotting for GPA and CD41 (Supplementary Figure S7). Purified EVs were then analyzed by LC-MS for their content in (i) ceramide, sphingomyelin and phosphatidylcholine, as major lipids enriched in lipid domains and mainly present in the outer leaflet or generated there; (ii) phosphatidylinositol, phosphatidylglycerol and phosphatidylethanolamine, as inner leaflet lipids; and (iii) lysophospholipids, which promote the formation of curved membrane due to their inverted cone-shape (Sobko et al., 2004; Lahdesmaki et al., 2010). In agreement with the decrease of ceramide-enriched domain abundance at the RBC membrane upon storage (see Figures 5A,C), ceramide and dihydroceramide species were enriched in RBC-derived EVs at the end of the storage period (Figures 6A,B). In contrast and in agreement with the increased abundance of sphingomyelin-enriched domains at the RBC surface upon storage, the main lipid species of those domains, i.e., sphingomyelin and phosphatidylcholine, were already at the detection limit at 1 week and further decreased upon storage (Figures 6C–E). The EV content in inner leaflet phosphatidylinositol and phosphatidylglycerol was globally decreased over the storage period, except for two species, whereas phosphatidylethanolamine species remained stable (Supplementary Figures S8A,C,D,F,G). In contrast to their precursors lysophosphatidylinositol and lysophosphatidylglycerol increased in RBC-derived EVs between the 3th and 4th weeks (Supplementary Figures S8B,E) whereas lysophosphatidylcholine and lysophosphatidylethanolamine species remained almost stable (Figure 6F and Supplementary Figure S8H).

### Longer ATP Preservation Does Not Restore Cholesterol-Enriched Domains, Delays ROS Accumulation and EV Release, Decreases the PS Exposure and SM- and GM1-Enriched Domain Rise and Abrogates the Increase of aSMase Activity and Calcium

**FIGURE 7:**
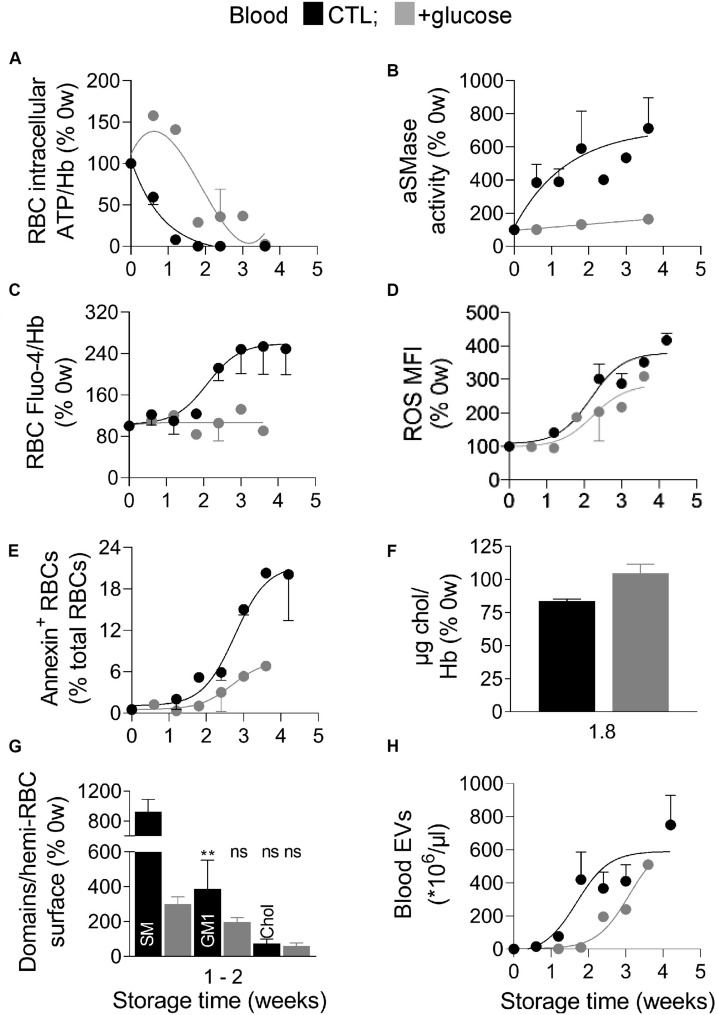
Blood glucose supplementation delays ATP drop, ROS accumulation and EV release, decreases the extent of PS exposure and of SM- and GM1-enriched domain increase and abrogates the increase of aSMase activity and calcium content. Blood samples were stored for the indicated times at 4°C in K^+^/EDTA tubes supplemented or not (black symbols) with a glucose-enriched solution (gray symbols) and measured for the intracellular ATP **(A)**, aSMase activity **(B)**, intracellular calcium and ROS contents **(C,D)**, PS surface exposure **(E)**, membrane cholesterol (chol) content **(F)**, lipid domain abundance **(G)**, and blood EVs **(H)**. **(A)** RBC ATP content determined as in Figure 1E. **(B)** aSMase activity evaluated as in Figure 4H. **(C)** RBC calcium content measured as in Figure 3A. **(D)** RBC ROS content assessed as in Figure 3C. **(E)** Proportion of PS-exposing RBCs determined as in Figure 4G. **(F)** RBC membrane cholesterol (chol) content, measured as in Figure 4A. **(G)** Sphingomyelin (SM), GM1- and cholesterol-enriched domain abundance, counted as in Figure 5. **(H)** Blood EV abundance, evaluated as in Figure 2C. Data are means ± SD of 1 **(A,C–E,H)** or 2 independent experiments [**B,F,G** (SM domains)] or means ± SEM of 3–4 independent experiments [**G** (GM1 and chol domains)]. Kruskal–Wallis test followed by Dunn’s multiple comparisons test to compare GM1- and chol -enriched domains at 0 weeks, 1–2 weeks – glucose and 1–2 weeks + glucose. ns, not significant; **p < 0.01.

The above data indicated that the RBC intracellular calcium and ROS contents, the membrane content in cholesterol, polyunsaturated FA and lipid peroxidation as well as the lipid transversal and lateral asymmetry were altered during storage of RBCs in K^+^/EDTA tubes. To next define the succession of events, the K^+^/EDTA tubes were supplemented directly after collection with an energy source, as in RBC concentrates (D’alessandro et al., 2010). As expected, such glucose addition delayed by 1.1 weeks the half-maximal decrease of intracellular ATP content and the complete loss of ATP to 3.6 weeks instead of 1.8 weeks (Figure 7A). In this condition, the aSMase activity, the intracellular RBC calcium content and the membrane cholesterol level were not modified at all upon storage (Figures 7B,C,F). In contrast, the intracellular ROS content, the PS surface exposure and the blood EV abundance were longer preserved upon energy supply but started to be modified from 1.8 weeks of storage, i.e., as soon as the RBC intracellular ATP content droped to ∼30% (Figures 7D,E,H). Finally lipid domains were differentially affected by the longer ATP preservation. For instance, sphingomyelin- and GM1-enriched domains were largely but not fully restored whereas cholesterol-enriched domains were not restored at all (Figure 7G). Three main observations were derived from those data. First, the oxidative stress appeared highly dependent to the intracellular ATP content and could represent the main contributor to the release of EVs. Second, the ATP level differentially influenced the abundance of lipid domains. Third, PS surface exposure during storage can be observed despite stable intracellular calcium content. These two last observations were further investigated by complementary pharmacological approaches.

### The Abundance of Sphingomyelin-Enriched Domains Is Specifically Increased Both Upon Acute ATP Depletion and aSMase Inhibition

**FIGURE 8:**
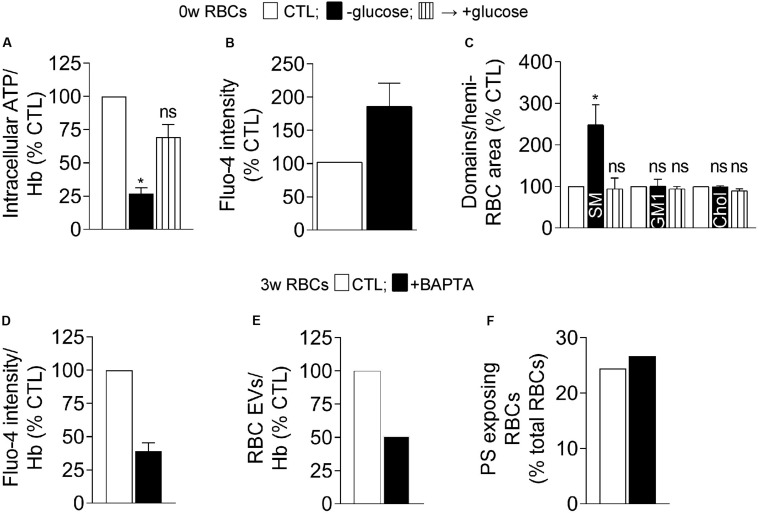
Acute ATP depletion in fresh RBCs increases the calcium content and the abundance of sphingomyelin-enriched domains whereas calcium chelation in 3 week-stored RBCs decreases the extent of vesiculation but not PS externalization. **(A–C)** Isolated fresh RBCs were incubated or not (open columns) in a medium devoid of glucose for 2 h to induce acute ATP depletion, followed or not (-glucose, black columns) by reincubation in a glucose-rich medium for 1 h for ATP repletion (+glucose, stripped black columns). RBCs were then analyzed for ATP content **(A)**, calcium content **(B)**, and abundance of lipid domains **(C)**. **(A)** RBC ATP content determined as in Figure 1E. Data were expressed in % of the CTL condition and are means ± SEM of 3 independent experiments (Friedman test followed by Dunn’s multiple comparisons test). **(B)** RBC calcium content. RBCs were labeled with Fluo-4-AM as in Figure 3A, immobilized on a silicon chamber and observed by fluorescence microscopy under destretching of the chamber for 7 min. Data were expressed in % of the CTL condition and are means ± SD of triplicates from 1 representative experiment out of two. **(C)** RBC lipid domain abundance counted as in Figure 5. Means ± SEM of 4–6 experiments (Wilcoxon signed rank test). **(D–F)** RBCs stored for 3 weeks at 4°C were isolated, incubated or not (CTL, open columns) with the intracellular calcium chelator BAPTA-AM (black columns) and analyzed for their calcium content **(D)**, EV production **(E)**, and PS surface exposure **(F)**. **(D)** RBC calcium content determined with Fluo-4-AM as in Figure 3A. Data were expressed in % of the CTL condition and are means ± SD from 2 independent experiments. **(E)** Abundance of EVs produced by RBCs. After incubation or not with BAPTA-AM, RBCs were pelleted to collect EV-containing supernatants which were submitted to ultracentrifugation to isolate EVs. EV abundance was then determined by Nanoparticle tracking analysis and normalized to the Hb content (as an estimation of the RBC abundance). Data were expressed in % of the CTL condition. **(F)** Proportion of PS-exposing RBCs determined as in Figure 4G. Data were expressed in % of total RBCs. ns, not significant; *p < 0.05.

To further test the dependency on ATP of the intracellular calcium content and the sphingomyelin-enriched domain abundance, fresh RBCs were acutely depleted in their ATP content. Upon ∼75% depletion by a 2 h-incubation in glucose-free medium, the intracellular calcium content was almost doubled (Figures 8A,B). Sphingomyelin-enriched domain abundance was significantly increased in contrast to GM1- and cholesterol-enriched domains whose abundance remained stable (Figure 8C). ATP repletion through addition of glucose-rich medium completely restored the number of sphingomyelin-enriched domains indicating non-toxicity of the treatment (Figures 8A,C). These results indicated that sphingomyelin-enriched domains can be modified by the intracellular ATP level, in agreement with observations in stored RBCs upon long term ATP decrease.

To then test the possible link between the aSMase activity and the abundance of sphingomyelin-enriched domains, RBCs were treated with amitriptyline (a functional inhibitor of this enzyme, Dinkla et al., 2012) and evaluated for sphingomyelin-enriched domains. Full aSMase activity inhibition specifically increased the abundance of sphingomyelin- but not GM1-enriched domains (Supplementary Figure S9), suggesting that sphingomyelin-enriched domains could be the target of aSMase upon storage.

### PS Surface Exposure Is Restored by Cholesterol Supplementation but Not by Calcium Chelation

To finally explore the apparent discrepancy between PS surface exposure and EV release during storage upon additional energy supply despite stable intracellular calcium content (see Figure 7C), 3 week-stored RBCs were treated with the intracellular calcium chelator BAPTA-AM. This treatment, which allowed to decrease the free calcium content by ∼65% (Figure 8D), declined by half the number of EVs produced by RBCs but did not affect the proportion of PS-exposing cells (Figures 8E,F). Those data again supported the absence of direct correlation between PS surface exposure, calcium increase and EV release by RBCs.

**FIGURE 9:**
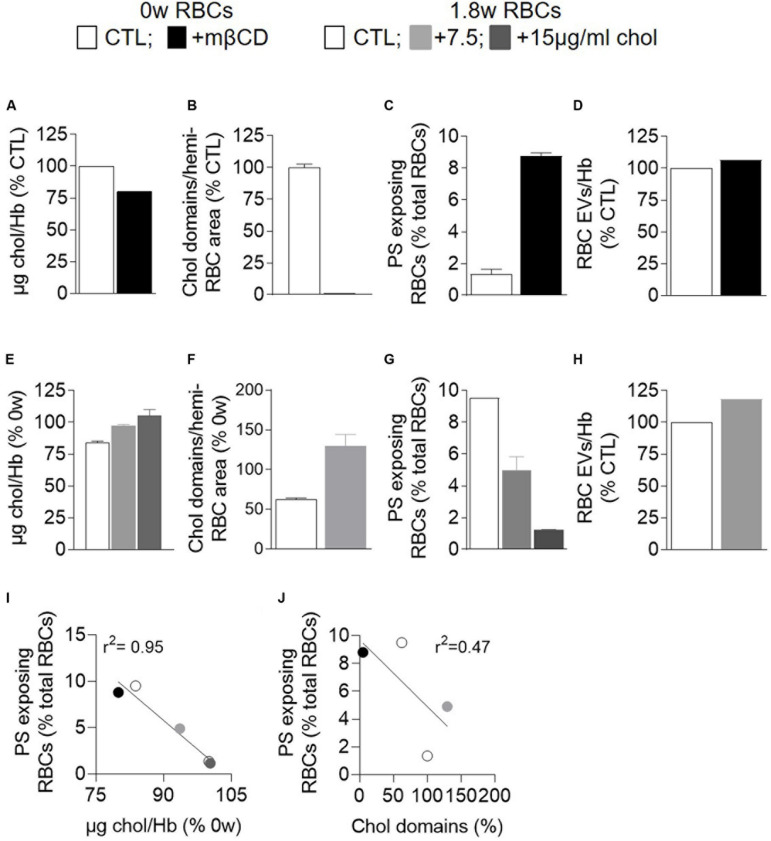
Modulation of the membrane cholesterol content correlates with the extent of PS surface exposure but not with the abundance of RBC EVs. Fresh RBCs were depleted in membrane chol with methyl-β-cyclodextrin (mβCD; **A–D,I,J**; black) whereas 1.8 week-stored RBCs were repleted with water-soluble chol at 7.5 or 15 μg/ml (**E–J**; light and dark gray). All RBCs were then analyzed for their chol content **(A,E)**, chol-enriched domain abundance **(B,F)**, PS surface exposure **(C,G),** and EV release **(D,H)**. **(A,E)** Chol content determined as in Figure 4A. Data are from Carquin et al. (2015) in **(A)** and are expressed as % of fresh untreated RBCs in **(E)** (means ± SD of 2 independent experiments). **(B,F)** Abundance of chol-enriched domains. Data were determined at in Figure 5 and are expressed in % of fresh untreated RBCs (means ± SD of 2 experiments). **(C,G)** PS exposure determined as in Figure 4G. Data are means ± SD of 2 independent experiments in **(C)** and of 1 experiment in duplicates in **(G)**. **(D,H)** EV released by RBCs determined as in Figure 8E. Data are expressed in % of untreated RBCs. **(I,J)** Correlation between PS surface exposure and the chol content **(I)** or the abundance of chol-enriched domains **(J)**.

We then tested whether cholesterol-related alterations could increase PS surface exposure. Indeed, cholesterol has been shown to inhibit the RBC scramblase activity at low intracellular calcium concentration (Arashiki and Takakuwa, 2017) and the cholesterol content and cholesterol-enriched domains were decreased upon storage before the free intracellular calcium started to increase (see above). To do so, fresh RBCs were incubated with mβCD to deplete ∼25% cholesterol (Figure 9A) and abolish cholesterol-enriched domains (Figure 9B). Whereas RBC vesiculation was not modified by this treatment, the proportion of PS-exposing RBCs was increased by ∼5-fold (Figures 9C,D), indicating that the alteration of cholesterol content and/or organization into domains could induce PS surface exposure in fresh RBCs and suggesting the implication of cholesterol in PS exposition upon storage.

To test the latter hypothesis, 1.8 week-stored RBCs were repleted with cholesterol through incubation with water soluble cholesterol at two different concentrations (Figure 9E). Such cholesterol repletion allowed to recover cholesterol-enriched domains to an even higher extent than on fresh RBCs (Figure 9F). The proportion of PS-exposing RBCs declined in a concentration-dependent manner, reaching at the highest concentration the level observed in fresh RBCs (Figure 9G), whereas the extent of RBC vesiculation was not modified (Figure 9H). Altogether, those data indicated that, as long as the intracellular calcium was low, surface PS exposure depended on the cholesterol content/organization into domains, as revealed by the correlation between those parameters (Figures 9I,J). However, as soon as the intracellular calcium increased, this correlation was no longer valid (data not shown), suggesting that other parameters such as calcium increase and ATP depletion also contributed to PS exposure.

## Discussion

### Evidence for the Modification of the RBC Plasma Membrane Lateral and Transversal Heterogeneity During Storage in K^+^/EDTA Tubes and Consequences for the RBC Physiology

Several lines of evidence provided in this study indicated that the RBC plasma membrane was strongly altered during storage in K^+^/EDTA tubes. First, the membrane lipid components were not randomly modified during storage, exhibiting a decrease of membrane cholesterol and linoleic acid at the benefit of long chain PUFA and a slight increase of lipid peroxidation. All those lipid modifications should contribute to affect the RBC morphology and functionality since (i) cholesterol and linoleic acid have all a rather fluidizing effect (Heron et al., 1980; Hochgraf et al., 1997), their decline being thus compatible with the increased membrane rigidity revealed by Laurdan and AFM; (ii) long chain PUFA are able to modulate Piezo1 channel (a mechano-activated ion channel) inactivation by decreasing membrane bending stiffness (Romero et al., 2019); (iii) the increased lipid peroxidation at the inner leaflet alters membrane packing and thickness (Wong-Ekkabut et al., 2007); and (iv) linoleic acid is known for its proinflammatory properties (Pereira et al., 2008; Innes and Calder, 2018). Second, all lipid domains were modified in abundance but not simultaneously neither at the same level nor by the same mechanism, as discussed below. For instance, cholesterol-enriched domains and cholesterol content were decreased very rapidly and simultaneously, which could be highly detrimental for RBCs through: (i) lower RBC deformability and increased elimination in the spleen, since cholesterol deficiency causes impaired RBC osmotic stability during *ex vivo* erythropoiesis (Bernecker et al., 2019) and cholesterol-enriched domains are necessary for the formation of high curvature areas during RBC deformation (Leonard et al., 2017); (ii) PS exposure and subsequent RBC elimination by macrophages, as revealed by the direct correlation between cholesterol content and the extent of PS exposure; and (iii) the increase of membrane rigidity, as revealed by Laurdan and AFM. Those modifications could in turn affect the dynamics of the low RBC curvature-associated domains enriched in GM1 or sphingomyelin, precluding calcium exchanges at the RBC surface (Conrard et al., 2018; Conrard and Tyteca, 2019). Hence, ceramide-enriched domains were lost upon storage, which could be favorable for the RBC as ceramide is known to be involved in several signaling pathways including cell death (Dinkla et al., 2012). Third, lipidomic analysis of EVs indicated their differential lipid enrichment/depletion upon storage, suggesting their shedding from lipid domains at the RBC surface. For instance, ceramide was enriched in EVs after 4 weeks but rather depleted before, in agreement with the late decrease of those domains from the cell surface. In contrast, sphingomyelin and phosphatidylcholine species, which were not expected to be lost from the cell surface based on the increased abundance of corresponding domains, were accordingly depleted in EVs upon storage. Fourth, the increased PS exposure during the first 2 weeks correlated with the level of membrane cholesterol while the later PS exposure correlated with the increased calcium concentration.

### Implication of the Modification of the Plasma Membrane Lateral Heterogeneity for RBC Vesiculation During Storage in K^+^/EDTA Tubes

We have previously proposed that lipid domains could represent platforms for vesicle biogenesis and shedding (Pollet et al., 2018). This hypothesis was based on the fact that domains are less ordered than the rest of the membrane, which causes a line tension and in turn induces vesiculation (Leonard et al., 2018). However, although all the types of domains are less ordered than the rest of the membrane (Conrard et al., 2018; Leonard et al., 2018), only those enriched in cholesterol and ceramide were able to vesiculate, suggesting a fine tuning of lipid domain dynamics at the RBC surface and specific triggering events for their vesiculation.

**FIGURE 10:**
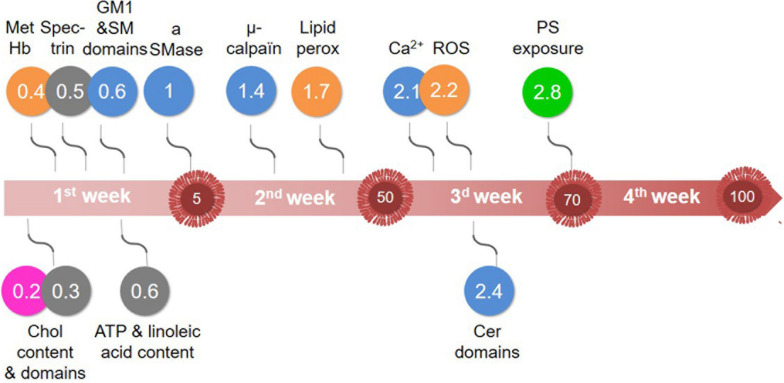
Major vesiculation-related events that take place during RBC storage in K^+^/EDTA tubes. Although the sequence of events may depend on the sensitivity of assays used, our data indicate alterations of: (pink) chol-enriched domain abundance; (orange) oxidative stress; (blue) calcium content and aSMase activity, with the increase of sphingomyelin-enriched domains and the decrease of cer-enriched domains; and (green) membrane transversal asymmetry, with the increase of PS surface exposure. Those events are initiated by the rapid decrease of the intracellular ATP content and modifications of the membrane lipid content and the spectrin membrane occupancy (gray). The large red arrow depicts the storage period analyzed in this study. The numbers highlighted inside red EVs indicate the number of EVs released by RBC at the end of each week, as estimated in Figure 2E. Events depicted above the arrow are increased by half of their maximal increase at the time indicated in the colored circles. Events depicted below the arrow are decreased by half of their maximal decrease at the time indicated in the circles. For additional explanations, see discussion.

For cholesterol-enriched domains (Figure 10, pink), this triggering event could be the increased membrane rigidity resulting from the alteration of both membrane lipid FA composition and membrane:cytoskeleton anchorage. Since cholesterol-enriched domains are particularly abundant in high curvature areas of resting RBCs where they exhibit the highest lipid order that can be found in lipid domains (Conrard et al., 2018) they represent the best target for EV release. This increased rigidity upon storage will create a line tension between the bulk membrane and cholesterol-enriched domains, which is then abrogated by vesiculation (Vind-Kezunovic et al., 2008). Those data indicate that EV shedding depends on cholesterol, in agreement with the hypothesis that the RBC membrane cholesterol content is declined by the release of cholesterol-enriched EVs (Santos et al., 2005) and with the increased number of EVs released upon low concentrations of mβCD to remove part of the membrane cholesterol (Gonzalez et al., 2009).

For sphingomyelin-enriched domains, the starting point could be the aSMase activation. Indeed, RBC exposure to the enzyme appeared to correlate with the release of EVs (Dinkla et al., 2012) and treatment with AMI (a functional enzyme inhibitor) is known to reduce vesiculation during storage (Hoehn et al., 2017). We showed here that the activity of aSMase increased in two stages during storage; first gradually, reaching at 4 weeks an activity six times higher than its initial activity; and then still three times more. This second peak of activity could be explained by the increased acidification of the plasma (Salzer et al., 2008) but this remains to be tested. Regarding the first increase, it correlated with the increased abundance of sphingomyelin-enriched domains and was followed by the decreased abundance of ceramide-enriched domains from the 3rd week of storage, suggesting that sphingomyelin clustering at the RBC surface represented a preferential target for aSMase activity (Figure 10, blue). However, the link between ceramide production and membrane blebbing is still unclear. Alterations of the membrane biophysical properties due to ceramide generation could partly provide this link, based on the following features. First, in agreement with (Fanani et al., 2009; Henry et al., 2013), we reported the ability of ceramide to form domains. Second, ceramide has a cone-shaped structure that can give a spontaneous curvature to the membrane. This property, combined with the fact that ceramide generated in the external leaflet may be redistributed to the inner one, could lead to membrane evagination (Lopez-Montero et al., 2005; Verderio et al., 2018).

The key question was whether the number of cholesterol- and ceramide-enriched domains lost could correspond to the number of EVs released per RBC each week. The abundance of EVs released per RBC rose from 5 after 1 week to ∼100 EVs after 4 weeks of storage (Figure 10, timeline), which is faster than in RBCs *in vivo* [up to 230 EVs during their 120 day-lifetime; Said and Doctor, 2017) and in RBC concentrates (Almizraq et al., 2013), mainly due to the absence of an energy source. Such fast RBC membrane area loss by vesiculation was supported by the increase of the spectrin occupancy per RBC area already after the 1st week of storage, which suggested spectrin-depleted EV release. Cholesterol-enriched domains decreased by ∼25% during the 1st week, which represented a loss of ∼4 domains per RBC, close to the 5 EVs measured at the end of the 1st week. Ceramide-enriched domains decreased in abundance during the 3rd week of storage, period during which ∼20 EVs were released by one RBC. Since those domains appeared to partially originate from the sphingomyelin-enriched domains which increased in abundance upon time, it is difficult to estimate the extent of their decrease. Thus, although lipid domain loss was compatible with a vesiculation-related process, they only represented a part of EVs released from RBCs, suggesting additional events, as discussed in the next two sections.

### Interplay With the Calcium-Based Model

We showed that calcium accumulated in RBCs upon storage in K^+^/EDTA tubes. As EDTA did not appear to completely chelate calcium in the extracellular medium, calcium accumulation in RBCs could result from calcium entry from the plasma. Furthermore, the plasma calcium concentration could be modified upon storage through calcium release by platelets and leukocytes upon death, eventually leading to calcium capture by RBCs. In terms of succession of events (Figure 10, blue), two events started to increase before calcium accumulates, i.e., the μ-calpain activity, which is not surprising based on its strong affinity for calcium (Bogdanova et al., 2013), and the PS surface exposure. Thus, PS exposure did not only depend on the calcium-dependent scramblase activation, as further supported by two other lines of evidence: (i) upon glucose addition to the blood tubes before storage, PS exposure was clearly visible despite normal intracellular calcium level; and (ii) PS exposure directly correlated with the membrane cholesterol content, as revealed by cholesterol depletion of fresh RBCs and cholesterol repletion of 1.8 week-old RBCs, in agreement with (Arashiki and Takakuwa, 2017). According to this model, cholesterol could act as a scramblase inhibitor, as a first indication of the interplay between the calcium-based model and membrane lipid alteration during storage.

The accumulation of the calcium itself occurred later, when ATP was no more detectable. Such accumulation therefore probably resulted from the shutdown of the plasma membrane calcium ATPase (PMCA) (Bogdanova et al., 2013). Moreover, several lines of evidence suggested that calcium accumulation also resulted from modifications of the biophysical properties of sphingomyelin-enriched domains during storage. We indeed previously showed the increased abundance of sphingomyelin-enriched domains during calcium efflux after RBC deformation. Since PMCA is the only calcium efflux pump in RBCs, we suggested at that time the implication of the sphingomyelin-enriched domains in the recruitment of PMCA and/or regulation of its activity. Since the aSMase activity increased during storage exactly after the increase of the abundance of sphingomyelin-enriched domains and because the inhibition of this enzyme by AMI also increased the abundance of these domains while decreasing the level of intracellular calcium (data not shown), we suggest that the modification of sphingomyelin-enriched domain biophysical properties (such as fluidity and curvature) through aSMase attack could alter the PMCA activity which requires an optimal environment in terms of fluidity (Tang et al., 2006). According to this model, the aSMase acts as a triggering event leading to sphingomyelin domain biophysical properties alteration, as a second indication of the interplay between the calcium-based model and membrane alteration during storage.

### Interplay With the Oxidative Stress-Based Model

We also confirmed the implication of the oxidative stress during storage. The first event detected was the oxidation of Hb into metHb followed by the oxidation of membrane lipids and ROS accumulation (Figure 10, orange). The fact that metHb accumulated before free ROS could result from the fact that under the storage conditions of tubes, the external O_2_ can diffuse. As a result, Hb is likely to be subject to a constant risk of auto-oxidation and as the antioxidant system becomes ineffective more quickly, metHb could accumulate without ROS at the beginning. In addition, Hb is both the source and the major target of these species. It could therefore serve as a buffer preventing their accumulation for a time. After 1 week of storage, the metHb did not increase anymore in contrast to free ROS, which could be due to the fact that metHb can be irreversibly transformed into hemichrome (Pauling and Coryell, 1936). While we did not measure the interaction between oxidized Hb and the membrane, such interaction could be reflected in the increase of the membrane stiffness during storage (Leonard et al., 2018). A long time after metHb production started the accumulation of ROS and the increase in lipid peroxidation which was surprisingly very low as compared to RBC concentrates (D’alessandro et al., 2012) a discrepancy that could result from the fact that we only measured malondialdehyde, one of the three products of lipid peroxidation besides isoprostanes and 4-hydroxynonenal (Janero, 1990).

The mechanism of oxidative stress appeared to act independently of the calcium accumulation and aSMase activity, as shown after supplementing the tubes with glucose. Nevertheless, since metHb increased before the first calcium-dependent event (i.e., the boost of calpain activity) and since acute ATP depletion (a condition not expected to lead to metHb accumulation) increased the abundance of sphingomyelin-enriched domains to a lower extent than upon similar ATP depletion obtained during storage, it is most than likely that the oxidative stress will influence the calcium- and aSMase-based models. This could occur through the accumulation of oxidized forms of Hb at the membrane (Kriebardis et al., 2007), and the resulting membrane cytoskeleton interaction alteration which could in turn increase the abundance of sphingomyelin-enriched domains, as previously shown (D’auria et al., 2013). Alternatively, oxidative stress could decrease the PMCA activity, as already shown in neurodegenerative diseases (Gorlach et al., 2015).

### Hypothetical Model for the Succession of Events Leading to RBC Vesiculation in K^+^/EDTA Tubes

Based on data in tubes supplemented or not with glucose, we propose the following model. The first event was the decrease of cholesterol-enriched domain abundance whatever glucose supplementation or not, suggesting an ATP-independent process (Figure 10, pink). The second event, which started to increase when the ATP was ∼30% and represented the main contributor to blood EV release, was the oxidative stress (Figure 10, orange). The third event was the calcium/aSMase. This model was less ATP-dependent but appeared related to the increase of sphingomyelin-enriched domains, the increased activity of aSMase and the alteration of sphingomyelin domain biophysical properties, leading to calcium accumulation and ceramide-enriched domain formation and vesiculation (Figure 10, blue). The fourth ultimate event occurred during the 4th week and was marked by PS externalization (Figure 10, green). In conclusion, we have shown that the modulation of lipid domains plays a significant role in the RBC vesiculation, releasing EVs of various lipid composition upon storage in tubes, but oxidative stress and PS exposure are also major events. The next step will be to test the relevance of those mechanisms to RBC vesiculation in blood bags before transfusion.

## Data Availability Statement

All datasets generated for this study are included in the article/Supplementary Material.

## Ethics Statement

The study was approved by the Medical Ethics Committee of the University of Louvain, Brussels, Belgium. The participants gave written informed consent.

## Author Contributions

A-SC and DT designed the experiments, analyzed and interpreted the data, and wrote the manuscript. MG and HP collected and analyzed the data. AS and JV were in charge of lipid imaging and quantification. ND assisted to flow cytometry experiments. LD’A assisted to nanoparticle tracking analysis. RT and GM were responsible for lipidomics on sorted EVs. EM and YL were responsible for fatty acid analysis on RBCs. PVDS did all the electron microscopy experiments. All the authors reviewed the final version of the manuscript.

## Conflict of Interest

The authors declare that the research was conducted in the absence of any commercial or financial relationships that could be construed as a potential conflict of interest.

## Supplementary Material

FIGURE S1 Fresh RBCs in K^+^/EDTA, heparin or citrate tubes exhibit similar intracellular ATP and calcium contents. Fresh isolated RBCs from K^+^/EDTA, heparin or citrate tubes were analyzed for their intracellular ATP **(A)** and calcium contents **(B,C)**. Data are expressed as % of K^+^/EDTA tubes. **(A)** Intracellular ATP content, determined as in Figure 1E. Means ± SEM of 4 independent experiments for heparin tubes (Mann–Whitney *U* test) and means ± SD of 2 independent experiments for citrate tubes. **(B)** Calcium content in heparin *vs.* K^+^/EDTA tubes, assessed as in Figure 3A. Means ± SD of triplicates. **(C)** Calcium content in citrate *vs.* K^+^/EDTA tubes. RBCs were labeled with Fluo-4 AM and analyzed by flow cytometry. The MFI of the RBC population was then determined. Data are means ± SEM of 4 independent experiments.

FIGURE S2 The global RBC intracellular hemoglobin content starts to decrease from 3 weeks of storage in K^+^/EDTA tubes. Global Hb content was determined by spectrophotometry at 450 nm upon storage to normalize contents in intracellular ATP **(A)**, intracellular calcium **(B)**, membrane chol **(C)** and membrane lipid peroxidation **(D)**. Data represented Hb absorbance values based on the quantity of RBCs engaged in each type of test (means ± SEM of 1–3 independent experiments).

FIGURE S3 Flow cytometry allows to distinguish EVs released by RBCs *vs.* those released by platelets. EVs were isolated from plasma by ultracentrifugation and labeled with CFSE, a general EV marker, and with fluorescent anti-GPA and anti-CD41 antibodies to identify EVs released by RBCs and platelets, respectively. EVs were increasingly diluted according to the storage time of the blood sample to avoid overcharge of the cytometer. The side scatter (SSC) *vs.* CFSE profiles and the anti-CD41-PE *vs.* anti-GPA-AlexaFluor647 profiles were gathered. Representative plots are shown.

FIGURE S4 Flow cytometry analysis upon storage reveals a single population of H_2_DCFDA-labeled RBCs with higher median fluorescence intensity which contrasts with distinct populations of Annexin V-labeled RBCs without fluorescence increase. RBCs were labeled with H_2_DCFDA to determine the intracellular ROS content **(A)** or with Annexin V-FITC to evaluate PS surface exposure **(B,C)** and analyzed by flow cytometry. **(A)** Representative plots of RBCs labeled with H_2_DCFDA upon storage. **(B)** MFI values determined with FlowJo of the global RBC population labeled with Annexin V-FITC (means ± SEM of 3 independent experiments). **(C)** Representative plots of RBCs labeled with Annexin V.

FIGURE S5 Membrane lipid order increases upon storage. Fresh (0w) or 2 week-stored RBCs (2w) were labeled with Laurdan, spread on PLL-coated coverslips and observed by vital multiphoton microscopy. Discocytes (disco) were distinguished from spherocytes (sphero) based on size and circularity. **(A)** Representative vital imaging. **(B)** Quantification of hemi-RBC area after 2 weeks of storage. **(C)** Quantification of membrane generalized polarization (GP) values for discocytes and spherocytes after 2 weeks of storage. Images are representative from 2 independent experiments and results are means ± SEM of 37–231 RBCs (one-way ANOVA followed by Tukey’s *post hoc* test). Adapted from Leonard et al. (2018).

FIGURE S6 Plasmatic pH levels are only very slightly decreased upon storage. Plasmatic pH levels were determined in 0–4 week-stored blood samples with the GEM PREMIER 3500.

FIGURE S7 RBC- and platelet-derived EVs are successfully sorted by magnetic force. EVs were isolated by ultracentrifugation from plasmas of K^+^/EDTA tubes stored for 0.9 and 1.3 weeks at 4°C. EVs released from RBCs were then sorted through incubation with magnetic beads coupled to anti-GPA antibodies by magnetic force. The sorted EVs were then analyzed by Western Blot for the presence of GPA to confirm successful sorting **(A)** and CD41 to exclude contamination **(B)**. A platelet lysate and EVs isolated from 1.3 weeks-stored tubes but not sorted were used as internal controls.

FIGURE S8 RBC-derived EVs are enriched in lysophosphatidylinositol and lysophosphatidylglycerol species but depleted in their precursors at the end of storage. RBC-released EVs were isolated from total blood EVs by immunopurification and analyzed by LC-MS for their content in: **(A,B)** phosphatidylinositol (PI) and lysoPI species; **(C–E)** phosphatidylglycerol (PG, further classified according to the length of their carbon chains) and lysoPG species; and **(F–H)** phosphatidylethanolamine (PE, further classified according to the length of their carbon chains) and lysoPE species. Each curve depicts one lipid species; circle, saturated lipid; triangle, lipid containing at least one MUFA; inverted triangle, lipid containing at least one PUFA; black to light gray colors, short to long carbon chains. All data are expressed as % of the EVs stored for 1 week and are means ± SD of 4 replicates from 1 experiment representative of 2.

FIGURE S9 Inhibition of the aSMase activity by amitriptyline specifically increases the abundance of sphingomyelin-enriched domains. Whole blood was incubated or not (CTL, open columns) with amitriptyline (+AMI, black columns) for 1h. Isolated plasmas were then tested for aSMase activity **(A)** while isolated RBCs were analyzed for the abundance of lipid domains **(B)**. **(A)** aSMase activity determined as in Figure 4H. Data are expressed in % of CTL. **(B)** Sphingomyelin (SM)- and GM1-enriched domain abundance determined as in Figure 5. Data are expressed in % of CTL (means ± SEM of 3–4 independent experiments; Wilcoxon signed rank test).
