# Supplementary figures and images for: Interplay Between Plasma Membrane Lipid Alteration, Oxidative Stress and Calcium-Based Mechanism for Extracellular Vesicle Biogenesis From Erythrocytes During Blood Storage
Source: Front Physiol. 2020 Jul 3;11:712. doi: 10.3389/fphys.2020.00712 (PMC7350142; doi:10.3389/fphys.2020.00712)

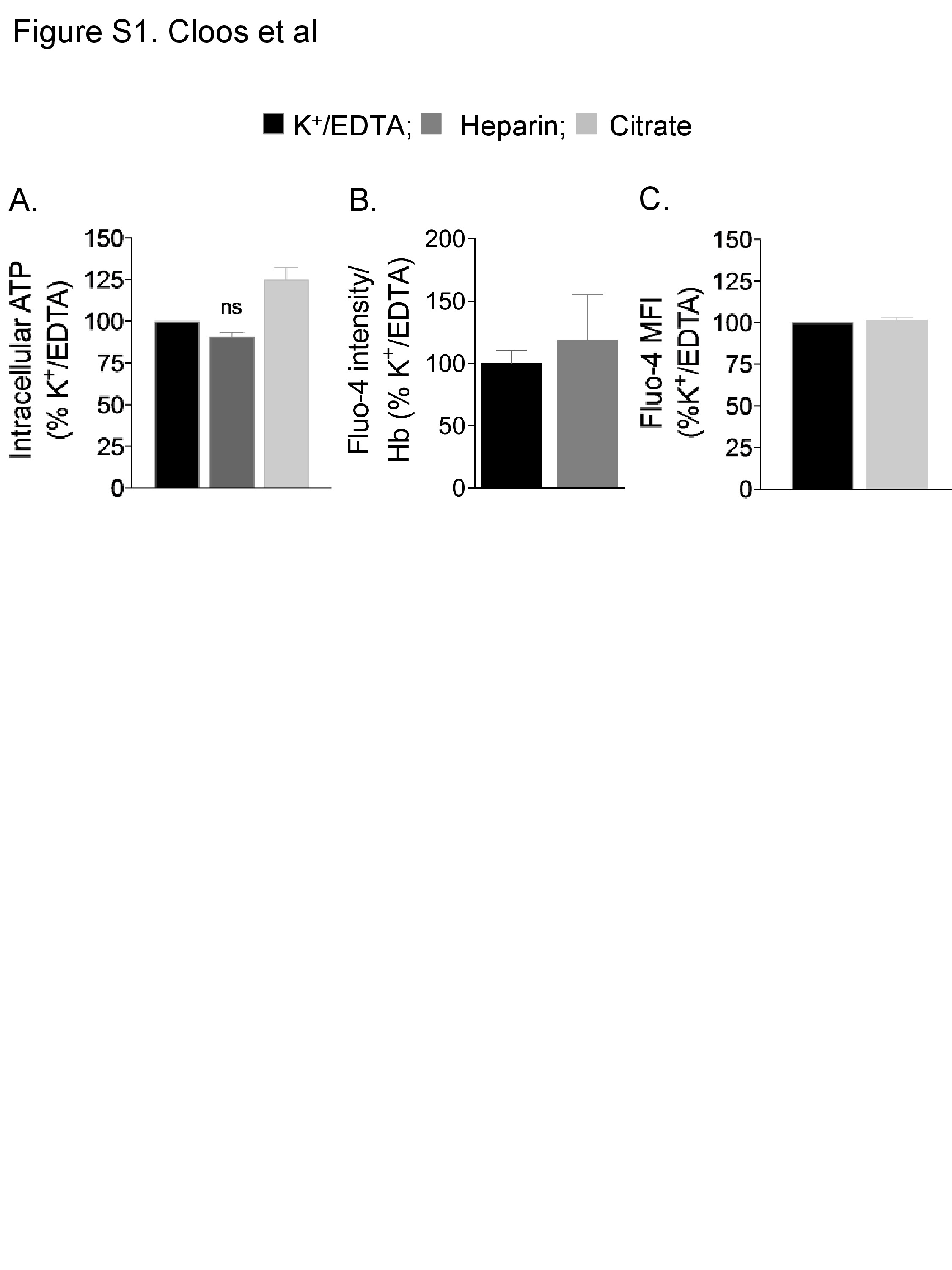

Supplement: FIGURE S1 — Fresh RBCs in K+/EDTA, heparin or citrate tubes exhibit similar intracellular ATP and calcium contents. Fresh isolated RBCs from K+/EDTA, heparin or citrate tubes were analyzed for their intracellular ATP (A) and calcium contents (B,C). Data are expressed as % of K+/EDTA tubes. (A) Intracellular ATP content, determined as in Figure 1E. Means ± SEM of 4 independent experiments for heparin tubes (Mann–Whitney U test) and means ± SD of 2 independent experiments for citrate tubes. (B) Calcium content in heparin vs. K+/EDTA tubes, assessed as in Figure 3A. Means ± SD of triplicates. (C) Calcium content in citrate vs. K+/EDTA tubes. RBCs were labeled with Fluo-4 AM and analyzed by flow cytometry. The MFI of the RBC population was then determined. Data are means ± SEM of 4 independent experiments. [file Image_1.JPEG]

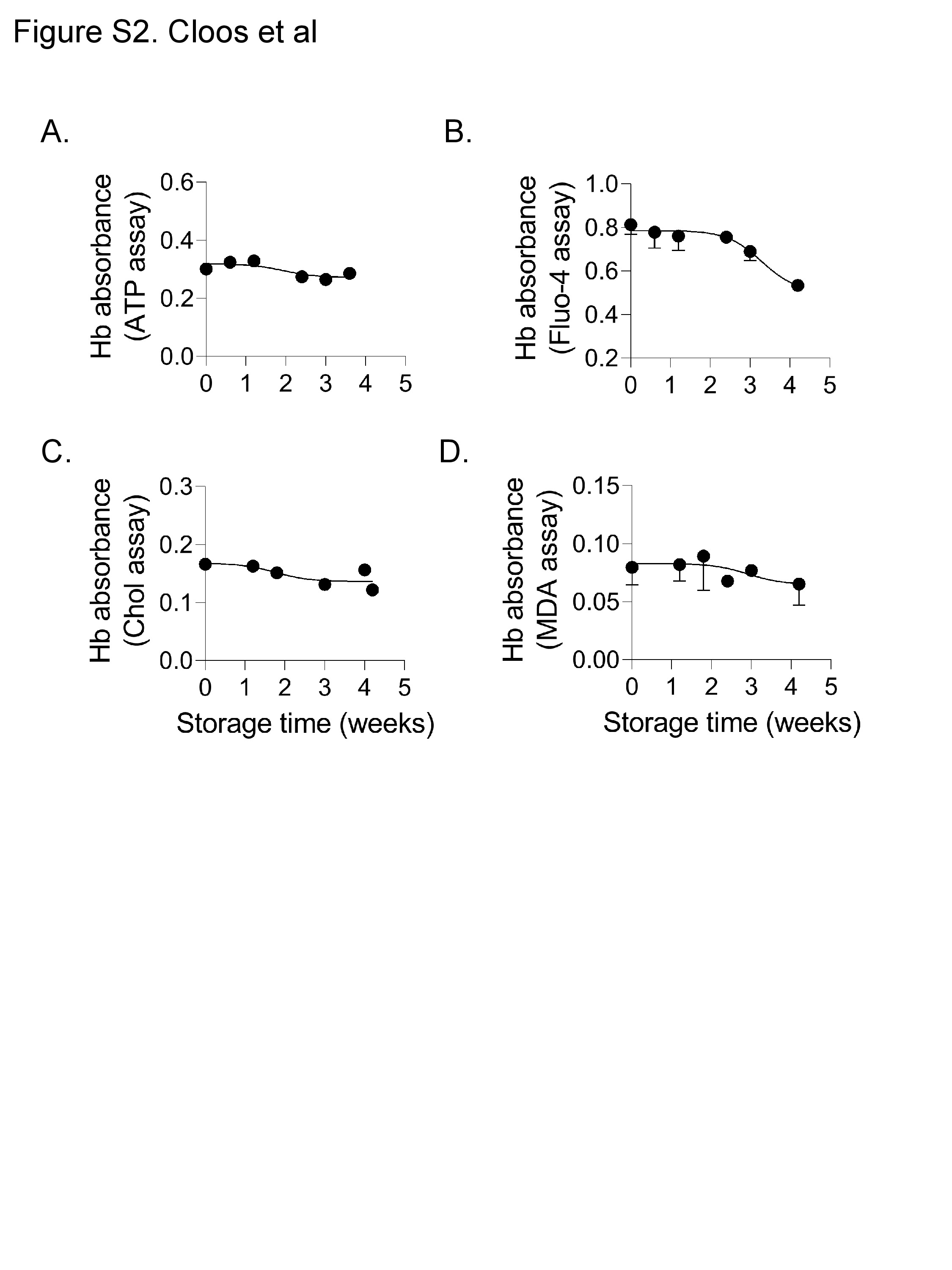

Supplement: FIGURE S2 — The global RBC intracellular hemoglobin content starts to decrease from 3 weeks of storage in K+/EDTA tubes. Global Hb content was determined by spectrophotometry at 450 nm upon storage to normalize contents in intracellular ATP (A), intracellular calcium (B), membrane chol (C) and membrane lipid peroxidation (D). Data represented Hb absorbance values based on the quantity of RBCs engaged in each type of test (means ± SEM of 1–3 independent experiments). [file Image_2.JPEG]

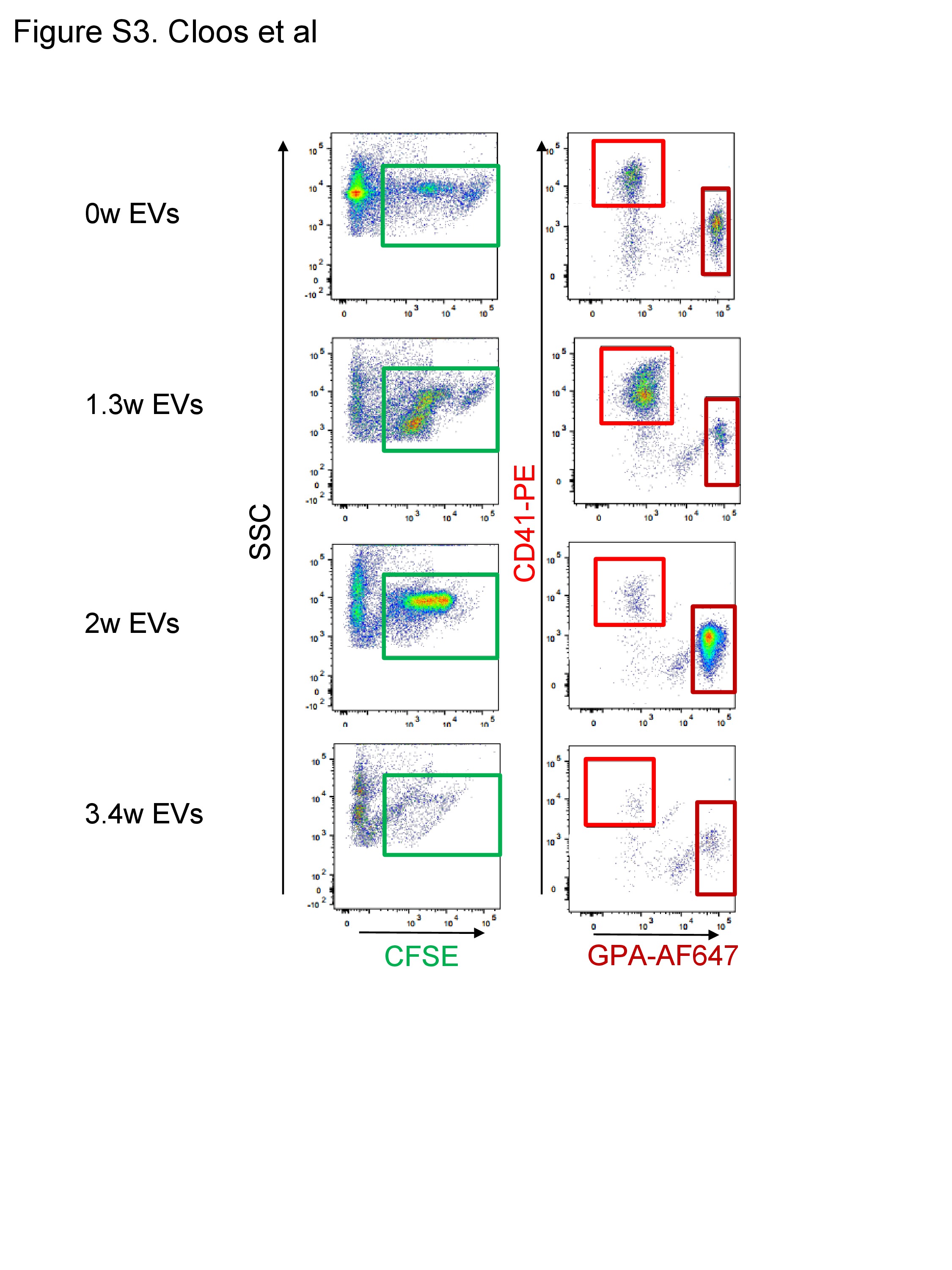

Supplement: FIGURE S3 — Flow cytometry allows to distinguish EVs released by RBCs vs. those released by platelets. EVs were isolated from plasma by ultracentrifugation and labeled with CFSE, a general EV marker, and with fluorescent anti-GPA and anti-CD41 antibodies to identify EVs released by RBCs and platelets, respectively. EVs were increasingly diluted according to the storage time of the blood sample to avoid overcharge of the cytometer. The side scatter (SSC) vs. CFSE profiles and the anti-CD41-PE vs. anti-GPA-AlexaFluor647 profiles were gathered. Representative plots are shown. [file Image_3.JPEG]

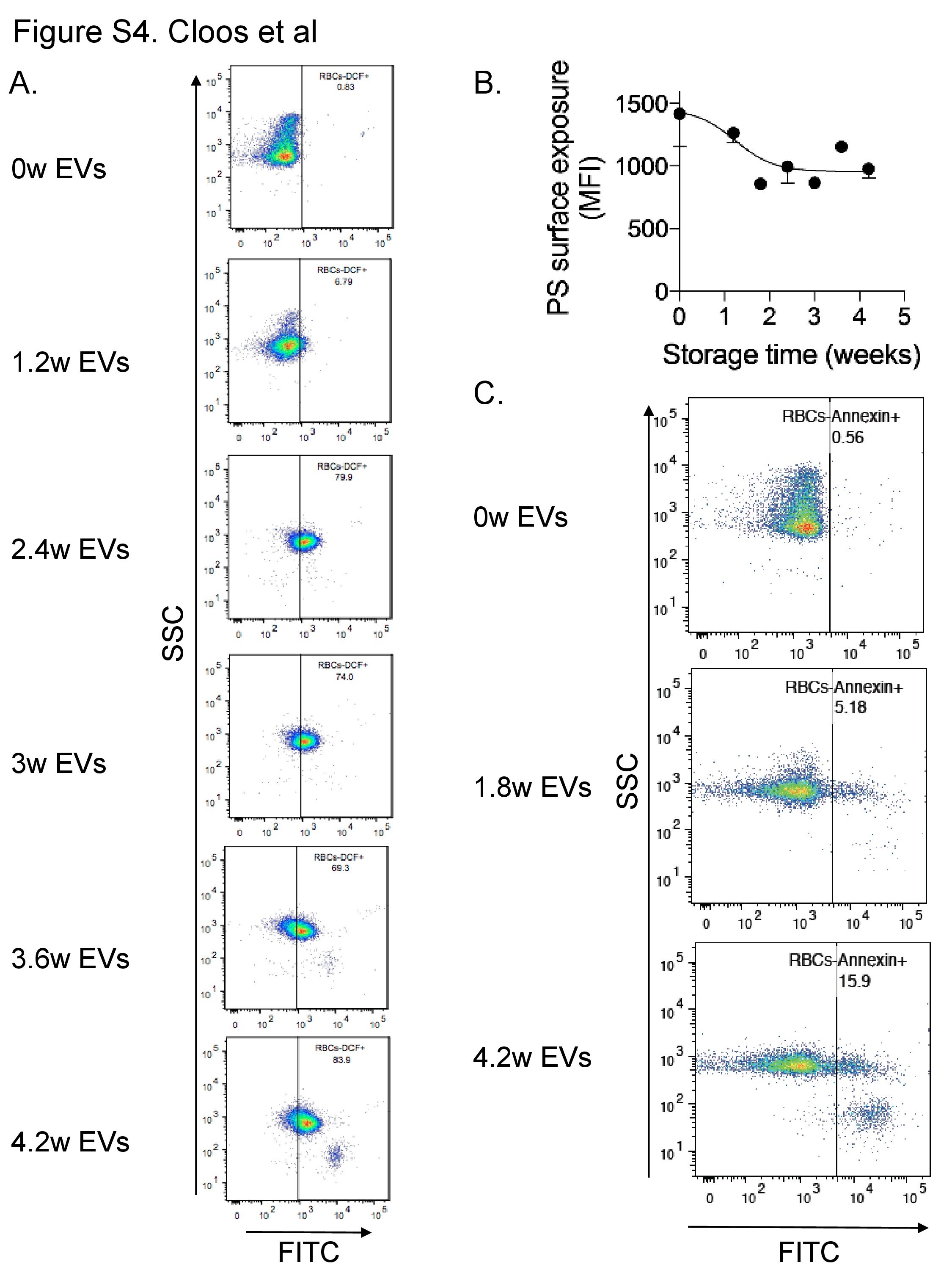

Supplement: FIGURE S4 — Flow cytometry analysis upon storage reveals a single population of H2DCFDA-labeled RBCs with higher median fluorescence intensity which contrasts with distinct populations of Annexin V-labeled RBCs without fluorescence increase. RBCs were labeled with H2DCFDA to determine the intracellular ROS content (A) or with Annexin V-FITC to evaluate PS surface exposure (B,C) and analyzed by flow cytometry. (A) Representative plots of RBCs labeled with H2DCFDA upon storage. (B) MFI values determined with FlowJo of the global RBC population labeled with Annexin V-FITC (means ± SEM of 3 independent experiments). (C) Representative plots of RBCs labeled with Annexin V. [file Image_4.JPEG]

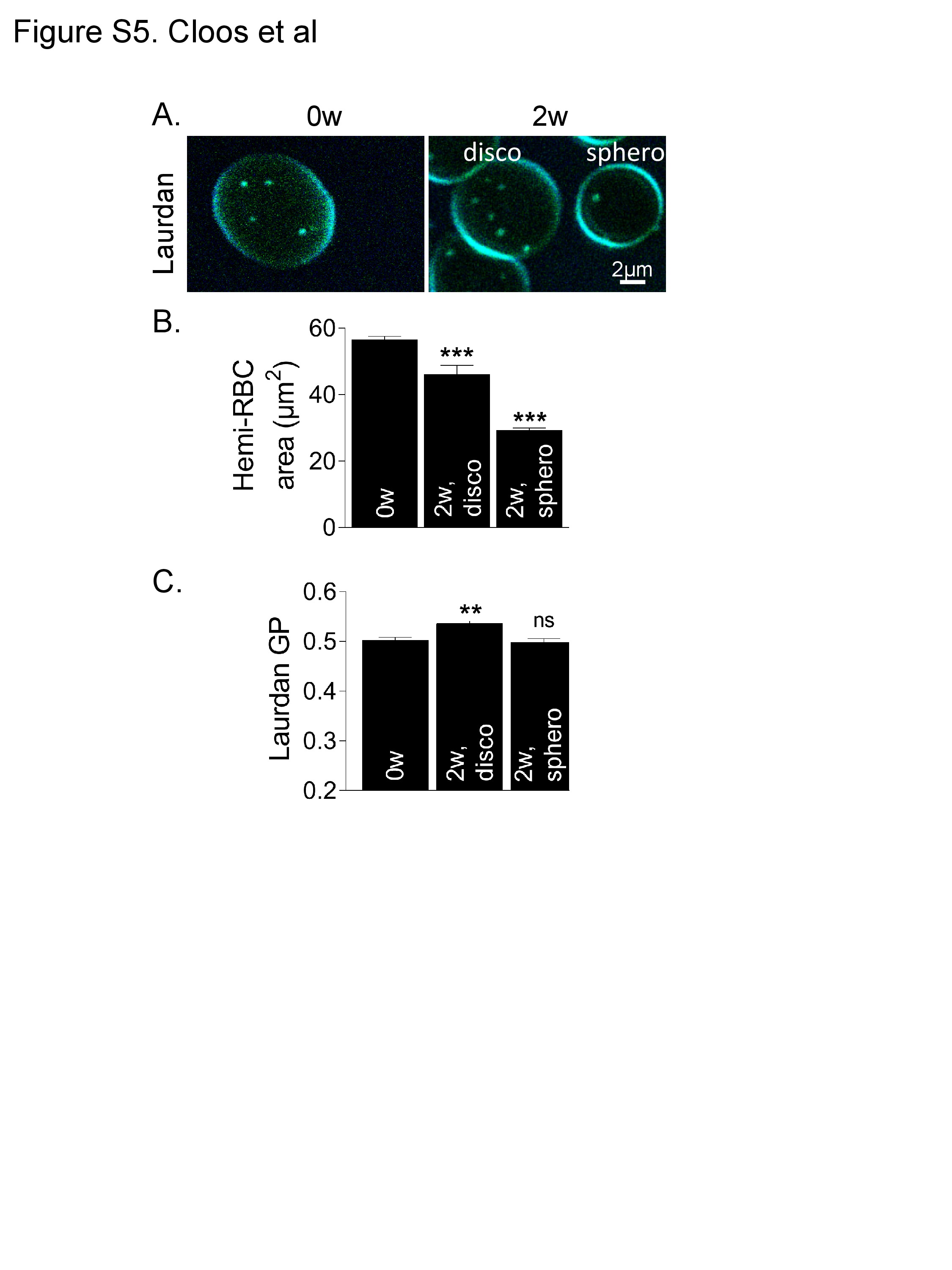

Supplement: FIGURE S5 — Membrane lipid order increases upon storage. Fresh (0w) or 2 week-stored RBCs (2w) were labeled with Laurdan, spread on PLL-coated coverslips and observed by vital multiphoton microscopy. Discocytes (disco) were distinguished from spherocytes (sphero) based on size and circularity. (A) Representative vital imaging. (B) Quantification of hemi-RBC area after 2 weeks of storage. (C) Quantification of membrane generalized polarization (GP) values for discocytes and spherocytes after 2 weeks of storage. Images are representative from 2 independent experiments and results are means ± SEM of 37–231 RBCs (one-way ANOVA followed by Tukey’s post hoc test). Adapted from Leonard et al. (2018). [file Image_5.JPEG]

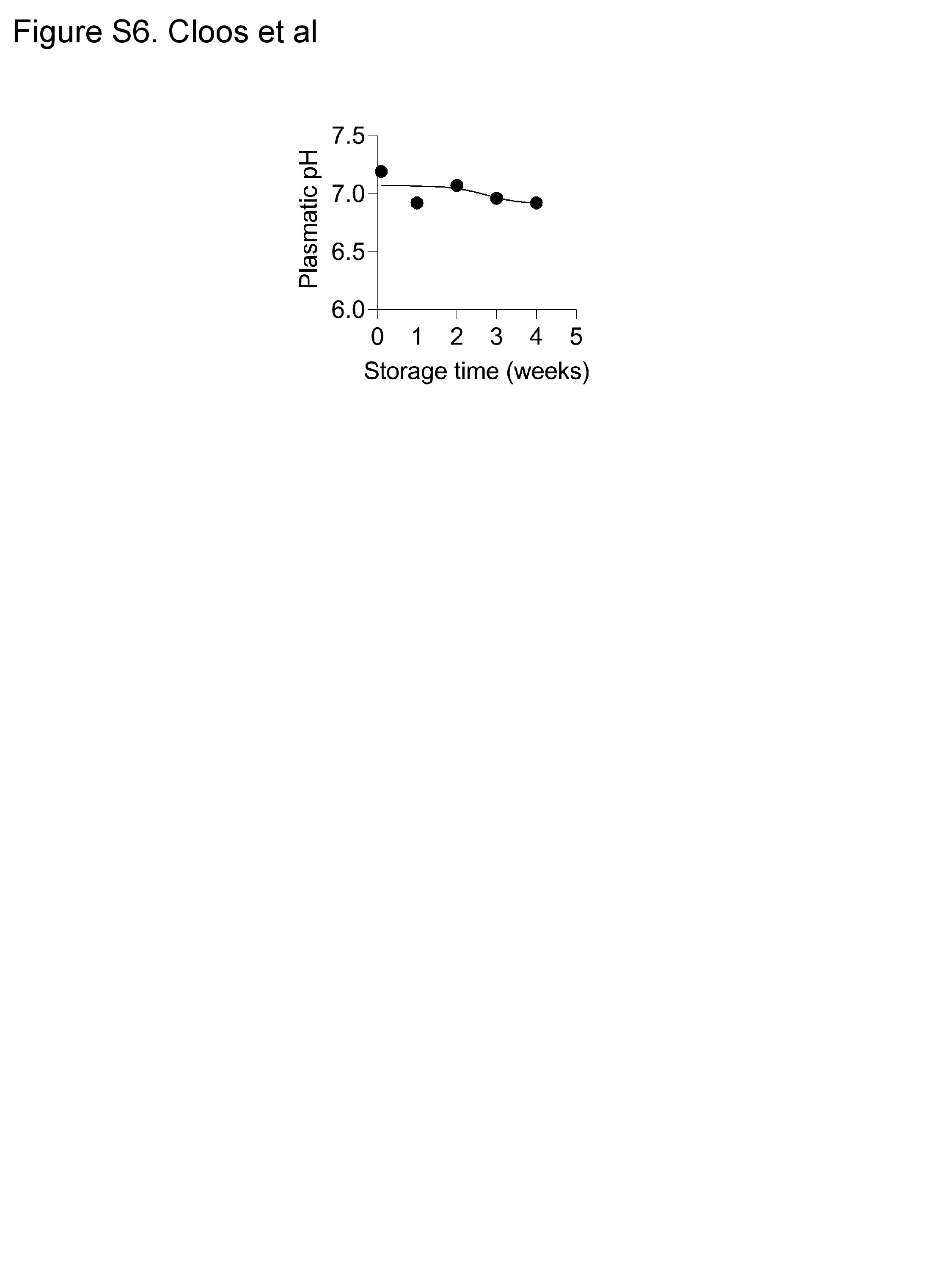

Supplement: FIGURE S6 — Plasmatic pH levels are only very slightly decreased upon storage. Plasmatic pH levels were determined in 0–4 week-stored blood samples with the GEM PREMIER 3500. [file Image_6.JPEG]

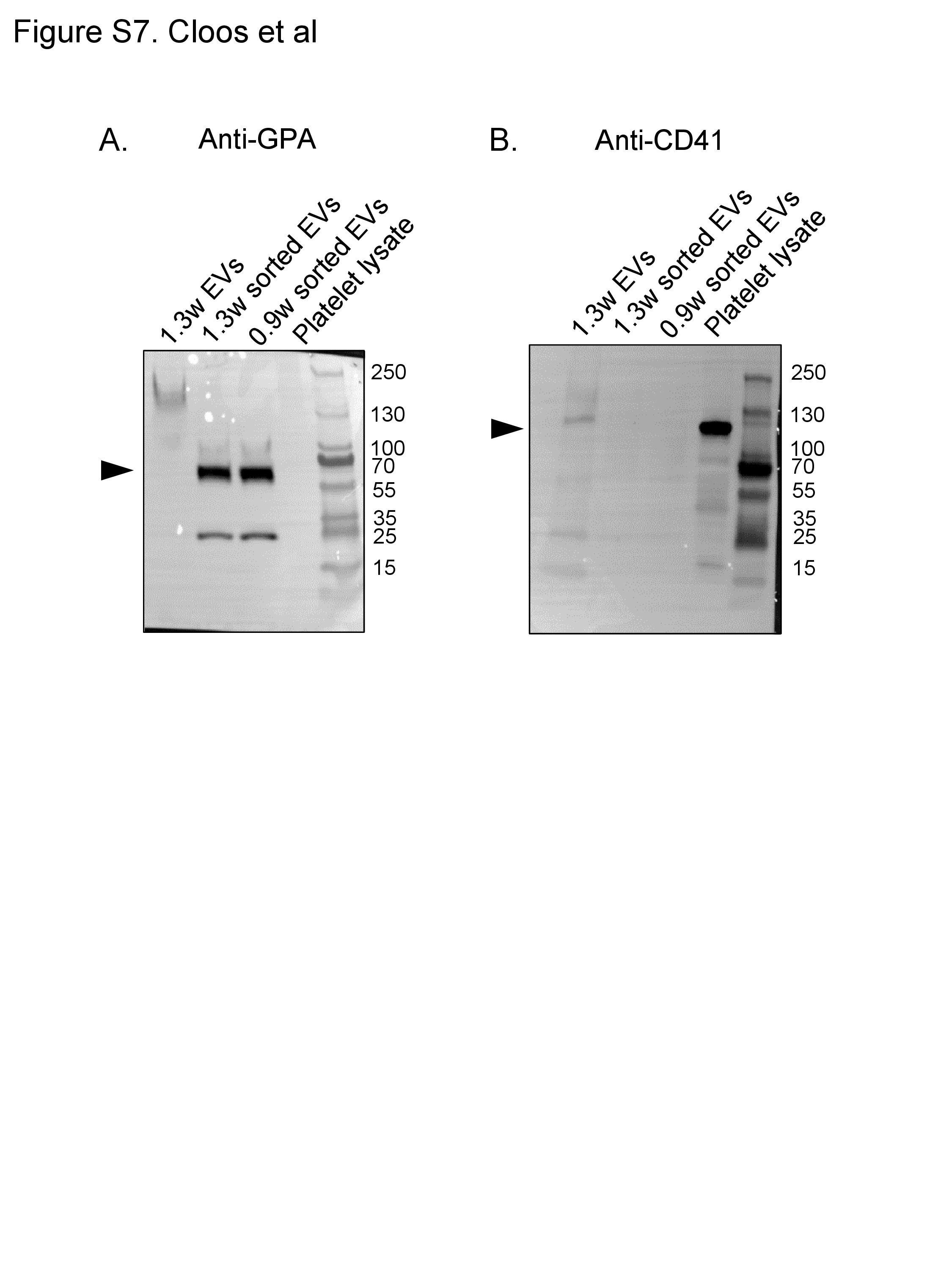

Supplement: FIGURE S7 — RBC- and platelet-derived EVs are successfully sorted by magnetic force. EVs were isolated by ultracentrifugation from plasmas of K+/EDTA tubes stored for 0.9 and 1.3 weeks at 4°C. EVs released from RBCs were then sorted through incubation with magnetic beads coupled to anti-GPA antibodies by magnetic force. The sorted EVs were then analyzed by Western Blot for the presence of GPA to confirm successful sorting (A) and CD41 to exclude contamination (B). A platelet lysate and EVs isolated from 1.3 weeks-stored tubes but not sorted were used as internal controls. [file Image_7.JPEG]

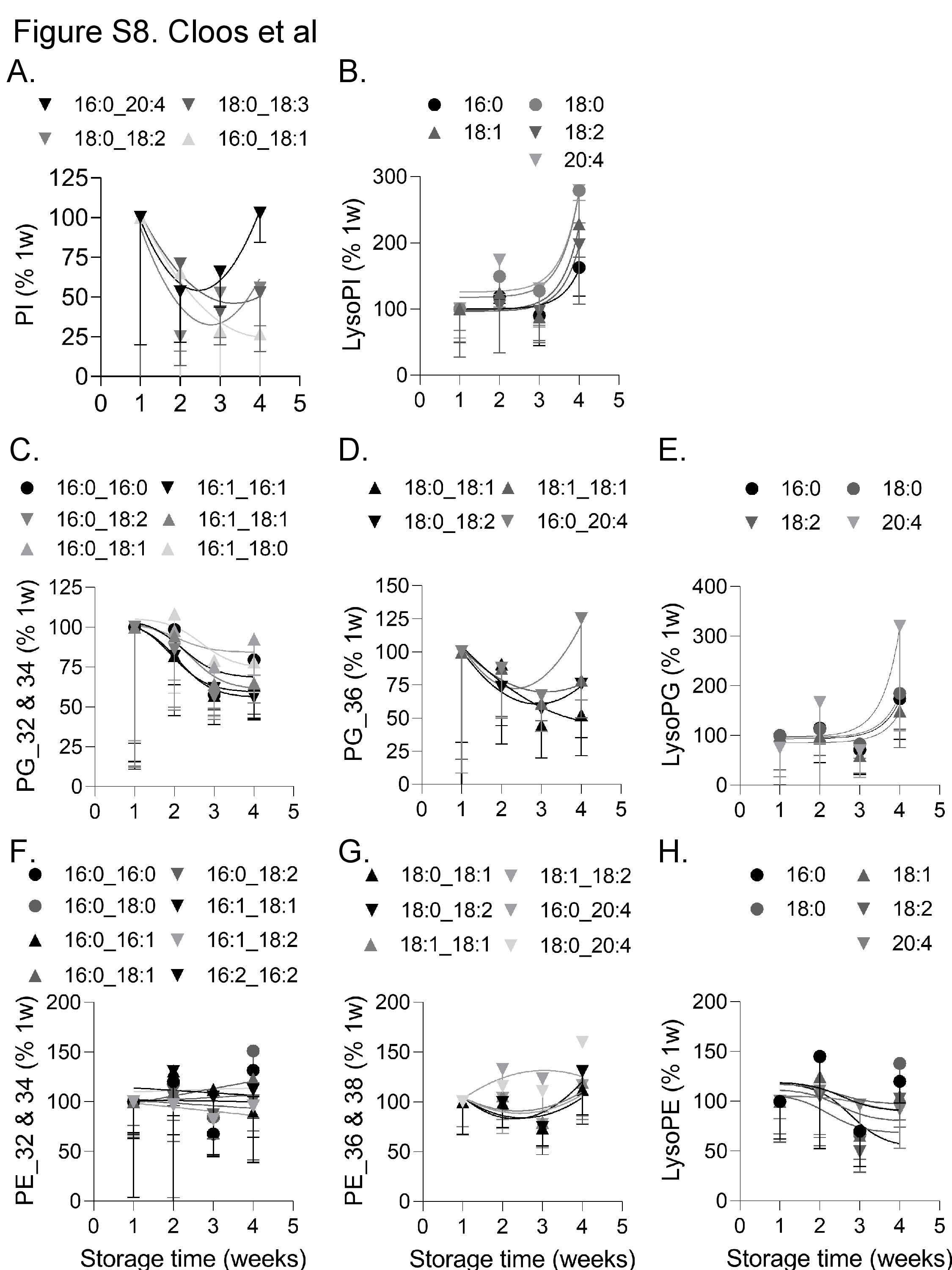

Supplement: FIGURE S8 — RBC-derived EVs are enriched in lysophosphatidylinositol and lysophosphatidylglycerol species but depleted in their precursors at the end of storage. RBC-released EVs were isolated from total blood EVs by immunopurification and analyzed by LC-MS for their content in: (A,B) phosphatidylinositol (PI) and lysoPI species; (C–E) phosphatidylglycerol (PG, further classified according to the length of their carbon chains) and lysoPG species; and (F–H) phosphatidylethanolamine (PE, further classified according to the length of their carbon chains) and lysoPE species. Each curve depicts one lipid species; circle, saturated lipid; triangle, lipid containing at least one MUFA; inverted triangle, lipid containing at least one PUFA; black to light gray colors, short to long carbon chains. All data are expressed as % of the EVs stored for 1 week and are means ± SD of 4 replicates from 1 experiment representative of 2. [file Image_8.JPEG]

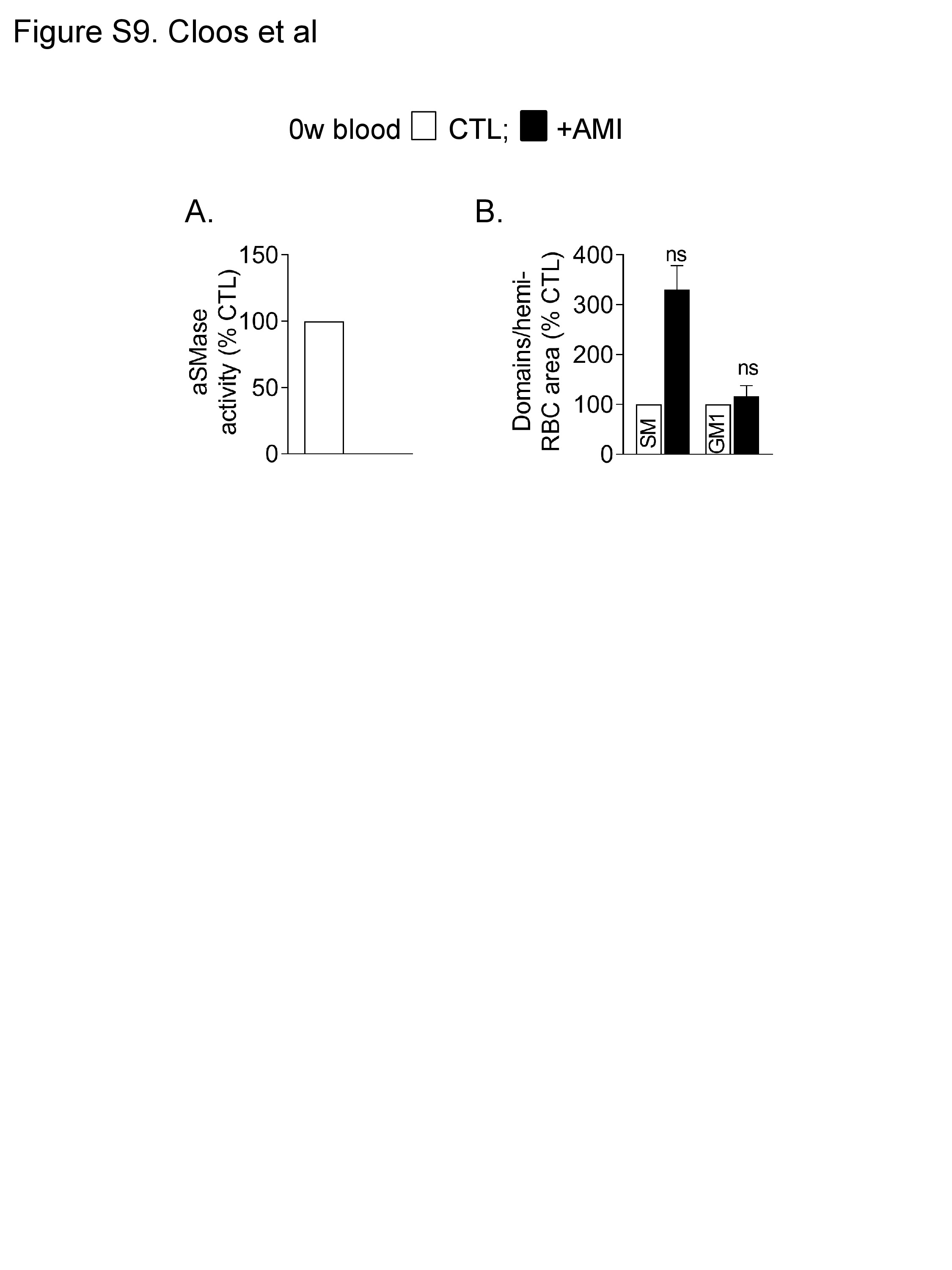

Supplement: FIGURE S9 — Inhibition of the aSMase activity by amitriptyline specifically increases the abundance of sphingomyelin-enriched domains. Whole blood was incubated or not (CTL, open columns) with amitriptyline (+AMI, black columns) for 1h. Isolated plasmas were then tested for aSMase activity (A) while isolated RBCs were analyzed for the abundance of lipid domains (B). (A) aSMase activity determined as in Figure 4H. Data are expressed in % of CTL. (B) Sphingomyelin (SM)- and GM1-enriched domain abundance determined as in Figure 5. Data are expressed in % of CTL (means ± SEM of 3–4 independent experiments; Wilcoxon signed rank test). [file Image_9.JPEG]
